# Advanced lyophilized mesenchymal stem cell-conditioned medium formulation improves therapeutic efficacy in skin wound repair

**DOI:** 10.1093/rb/rbag140

**Published:** 2026-06-29

**Authors:** Xinjiani Chen, Bailei Li, Wenxue Gu, Mingjuan Li, Zhen Zhang

**Affiliations:** Department of Pharmaceutics, College of Medicine, Jiaxing University, Jiaxing 314001, China; Department of Biotechnology and Biomedicine, Yangtze Delta Region Institute of Tsinghua University, Jiaxing 314006, China; Zhejiang Key Laboratory of Multiomics and Molecular Enzymology, Yangtze Delta Region Institute of Tsinghua University, Jiaxing 314006, China; Department of Biotechnology and Biomedicine, Yangtze Delta Region Institute of Tsinghua University, Jiaxing 314006, China; Zhejiang Key Laboratory of Multiomics and Molecular Enzymology, Yangtze Delta Region Institute of Tsinghua University, Jiaxing 314006, China; Department of Pharmaceutics, College of Medicine, Jiaxing University, Jiaxing 314001, China; Department of Biotechnology and Biomedicine, Yangtze Delta Region Institute of Tsinghua University, Jiaxing 314006, China; Zhejiang Key Laboratory of Multiomics and Molecular Enzymology, Yangtze Delta Region Institute of Tsinghua University, Jiaxing 314006, China

**Keywords:** mesenchymal stem cells, conditioned medium, lyophilization, cryoprotectants, skin wound healing

## Abstract

The secretome of human umbilical cord mesenchymal stem cells (hUC-MSCs) represents a promising cell-free therapeutic approach for wound healing because of its rich composition of bioactive factors that promote tissue repair. However, the clinical translation of secretome-based therapies is hindered by the instability of its bioactive components during storage. To address this challenge, we developed a lyophilization strategy using cryoprotectants to produce a stable, powdered form of hUC-MSC-conditioned medium (CM). Our results demonstrated that the lyophilized CM (Lyo-CM), particularly when formulated with specific cryoprotectants, maintained excellent bioactivity of CM while showing superior reproducibility and stability. Furthermore, both *in vitro* and *in vivo* experiments revealed that Lyo-CM significantly enhanced wound closure during inflammation and proliferation phases, including inflammation resolution, cell proliferation, angiogenesis, and extracellular matrix remodeling. Our study provides a valuable approach to establishing a clinically viable, shelf-stable formulation of hUC-MSC secretome to advance the field of cell-free regenerative therapy.

## Graphical abstract

**Graphical Abstract:**
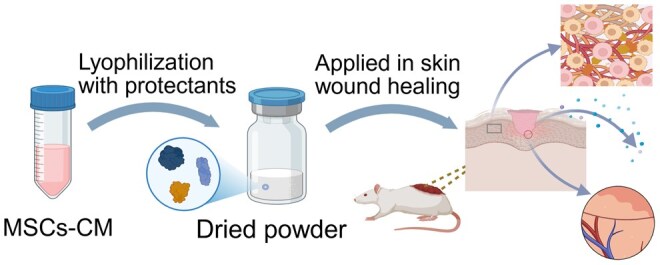

## Introduction

Cutaneous wound healing, a multifaceted biological process encompassing hemostasis, inflammation, proliferation, and remodeling [1], remains a critical clinical challenge due to risks of an extended trajectory, infections, and impaired recovery. Mesenchymal stem cells (MSCs), also referred to as mesenchymal stromal cells, have emerged as promising regenerative agents over the past two decades [2–4], owing to their multidifferentiation potential and immunomodulatory properties [5, 6]. However, direct MSC application to wounds comes with limitations that include immunogenicity and ethical concerns. Recent studies have elucidated that MSC-derived therapeutic effects are largely mediated by paracrine signaling [7–9], shifting focus toward cell-free alternatives such as MSC-derived exosomes (MSC-Exos) [10–12] and MSC-derived conditioned medium (MSC-CM) [13–17].

MSC-CM is referred to as the stromal cell culture medium that has removed the cells and comprises the secretomes such as growth factors, chemokines, cytokines, and extracellular vesicles (EVs) [18, 19]. In contrast with MSC-Exos, MSC-CM retains a broader array of bioactive components and does not require a complex production process. Previous studies on MSC-CM have demonstrated reduced inflammation at the early stage of wound healing [20]; promotion of regenerative epithelialization through enhanced proliferation and migration of endothelial cells [21, 22], fibroblasts [23, 24], and keratinocytes [25, 26]; upregulated synthesis of extracellular matrix (ECM) [27, 28]; enhanced neovascularization [29]; and promotion of epithelial-mesenchymal transition [30]. Despite these benefits, clinical translation of MSC-CM is impeded by challenges such as inconsistent formulation protocols, undefined bioactive composition, and product degradation during storage [14, 31].

At present, the preservation of MSC-CM primarily depends on conventional freezing at −20°C or −80°C [27, 32, 33]. This low-temperature storage strategy imposes strict logistical limitations on sample transportation and fails to sustain the long-term structural and functional stability of cryo-sensitive bioactive components in MSC-CM, particularly EVs [34, 35]. As a promising alternative preservation technology, lyophilization removes moisture from samples and reduces structural degradation, enabling the long-term storage of MSC-CM [36]. Nevertheless, freeze-drying also introduces mechanical stresses (e.g., ice crystallization, dehydration) that may destroy bioactive properties of constituents [37]. The incorporation of cryoprotectants has been proven as an effective approach to alleviating lyophilization-induced structural damage [38]. Nonreducing disaccharides such as trehalose and mannitol have already been confirmed to protect the morphology of EVs [39]. Despite these advances, their utility in preserving MSC-CM bioactivity remains insufficiently explored [40], with limited data on how protective agents influence functional outcomes in wound healing.

This work focuses on two representative cryoprotectants with distinct protective mechanisms, namely trehalose and PVP40, to develop an optimized lyophilization formulation for MSC-CM preservation. Trehalose is a nonreducing disaccharide with a high-glass transition temperature (*T*_g_) and low hygroscopicity, which stabilizes biomolecules by forming an amorphous glassy matrix that minimizes molecular mobility and ice crystal nucleation while avoiding Maillard reactions [39, 41]. In contrast, PVP40 is a nonionic polymer with osmotic inertness, which differs from the water-replacement and osmotic regulatory mechanism of trehalose. It synergistically enhances cryoprotective efficacy via steric hindrance on biomolecular surfaces and disruption of ice lattice assembly, thereby complementing the protective function of trehalose. This unique synergistic effect endows the combined formulation with great potential to comprehensively protect diverse bioactive components in MSC-CM.

Accordingly, this study aimed to investigate the protective effects of trehalose combined with PVP40 on the structural and functional degradation of bioactive factors in MSC-CM during lyophilization. We systematically characterized the compositional stability, nanoparticle integrity, and biological activity of MSC-CM preserved with single or compound cryoprotectants. Furthermore, the therapeutic potential of optimized lyophilized MSC-CM was validated both *in vitro* and in a rat full-thickness skin wound model, with a particular focus on its regulatory effects on the inflammatory and proliferative stages of wound healing. Collectively, this work demonstrated that the dual cryoprotectant-stabilized lyophilized MSC-CM served as a stable, long-term storage-compatible cell-free therapeutic platform for skin wound regeneration, providing a feasible strategy to advance the translational application of MSC-CM-based regenerative therapies.

## Materials and methods

### Culture and identification of human umbilical cord mesenchymal stem cells

Human umbilical cord mesenchymal stem cells (hUC-MSCs) were obtained from Jiaxing Regional Cell Preparation Center. MSCs at passages 3-5 (P3-5) were cultured in serum-free medium (#RP02010, Anhui Shownin Biology, China) under standard culture conditions (37°C, humidified atmosphere composed of 95% air / 5% CO_2_). All procedures were performed under aseptic conditions in a GMP-certified facility using class II biosafety cabinets and the cells showed no mycoplasma infection.

For large-scale cell culture and conditioned medium collection, MSCs were seeded in a scalable cell factory system (Thermo Scientific™ Nunc™ EasyFill™-2 Cell Factory™, Product # 169171) with a cell density of 4 × 10^3^ cells/cm^2^. Each unit provided a total cultivation area of 1261 cm^2^. Cell identification was performed in strict accordance with the phenotypic criteria established by the International Society for Cellular Therapy. Flow cytometry analysis of typical MSC surface markers (positive: CD73, CD90, CD105; negative: CD34, CD45, CD11b, CD19, HLA-DR) was completed by a professional commercial testing institution. In addition, the multilineage differentiation potential of hUC-MSCs was verified via osteogenic, adipogenic, and chondrogenic induction, followed by Alizarin Red S, Oil Red O, and Alcian Blue staining, respectively. Detailed identification protocols are provided in Supplementary Material S1, and the obtained results confirmed the standard biological characteristics of hUC-MSCs (Supplementary Figure S1).

Cell supernatant was harvested from MSCs when they reached 80–90% confluence (every 4 days postseeding). Under these conditions, ∼150 mL of raw supernatant was collected per cell factory.

### Preparation and lyophilization of conditioned medium

The collected cell supernatant was first centrifuged at 500*g*, 4°C for 5 min and then centrifuged at 3000*g*, 4°C for 10 min to eliminate any cell contamination. Lastly, the fresh supernatant was filtered through a 0.22-µm PES membrane filter (#421097, Corning, USA), and after subpackaging, all were stored at −80°C. This collection is referred to as “Original CM” throughout the manuscript.

Different cryoprotectants were used for the lyophilization process. Trehalose (#T0167, Sigma-Aldrich, USA) was dissolved in phosphate-buffered saline (PBS)/double-distilled water (65:35 v/v) to prepare solutions at 50–200 mM. PVP40 (Sigma-Aldrich, USA) was added to select trehalose solutions at a 2% (w/v) final concentration to achieve an osmotic pressure that approximated that of extracellular fluid [42].

Sterilized cryoprotectant solutions (autoclaved at 121°C, 15 min) were stored at 25°C in light-protected vials. For lyophilization, Original CM was combined 1:1 (v: v) with protectant solution. All samples were rapidly frozen at −80°C for 12 h as the prefreezing step, and subsequently transferred into a desktop freeze dryer (LC-10N-50, Lichen, China). The formal lyophilization procedure was performed under a continuous vacuum chamber pressure of 10–15 Pa, with a shelf temperature maintained at **−**50°C for 24 h. After completion of lyophilization, three types of lyophilized CM samples were obtained, including CM0 (without cryoprotectant), CM1 (trehalose only), and CM2 (trehalose + PVP40 combination). In subsequent experiments, each lyophilization tube contained exactly 1.0 mL of the Original CM for consistent processing and comparison across all experimental groups.

The lyophilized CM variants were rehydrated with cell culture medium or double-distilled water (ddH_2_O) at predefined dilutions for functional assays.

### Osmolality measurement

The osmotic pressure of different CMs (Original CM, various concentrations of trehalose and PVP40) were measured using a freezing point osmometer (OsmoTECH XT, Advanced Instruments, USA) in duplicate and the values were averaged. The osmotic pressure of different concentrations of trehalose and PVP40 are specified in Supplementary Figure S2. The ideal protectant solution was classified as having an osmotic pressure comparable to that of the Original CM.

### BCA protein assay

The protein concentrations of different batches of CM were quantified using a bicinchoninic acid (BCA) assay kit (#PC0020, Solarbio, China). Samples were diluted in PBS to fall within the linear range of a standard curve created by a BSA standard. Absorbance was measured at 562 nm using an EnSight multimode plate reader (PerkinElmer, USA). All measurements were performed in triplicate to ensure reproducibility. Total protein content was expressed as mg/mL.

### Nanoparticle tracking analysis

Particle concentrations and hydrodynamic diameters within conditioned medium were quantified using a Particle Metrix ZetaView nanoparticle tracking analyzer (ZetaVIEW, Particle Metrix, Germany) operated via ZetaView software (v8.05.14 SP7). The instrument was precalibrated using 100 nm polystyrene reference particles (1× PBS suspension) and flushed with PBS between measurements. Samples were diluted 1:200 in PBS and triplicate recordings were performed at 30°C with 11 sequential fields of view per sample analyzed to ensure statistical robustness.

### Enzyme-linked immunosorbent assay kits for component analysis

The cytokine composition was quantitatively analyzed across four variants of the conditioned medium: Original CM, CM0, CM1, and CM2. Key inflammatory cytokines (IL-6, IL-10, MCP-1, CXCL-9) and growth factors (TGF-β1, vascular endothelial growth factor [VEGF], keratinocyte growth factor [KGF], fibroblast growth factor [FGF], hepatocyte growth factor [HGF], epidermal growth factor [EGF]) were quantified using human-specific enzyme-linked immunosorbent assay (ELISA) kits. Reagent manufacturers and catalog numbers are detailed in Supplementary Table S1. Assays were performed according to the manufacturer’s instructions. Technical triplicate measurements were conducted and averaged per group. Final protein concentrations were derived from the standard curve.

### Scanning electron microscopy and energy-dispersive X-ray spectroscopy

The morphology of each lyophilized composition was investigated using scanning electron microscopy (SEM; JSM-7800F, JEOL, Japan). Each group of lyophilized powder was fixed to a carbon tape and then subjected to gold coating. The morphology scanning was performed ranging from 5 to 15 kV without damage to the samples. Additionally, energy-dispersive X-ray spectroscopy (EDS) was performed to identify the properties of powders at 8 kV.

### Transmission electron microscopy

Fresh and lyophilized MSC-CM samples were concentrated by ultrafiltration. Briefly, lyophilized sample was redissolved with 1 mL ddH_2_O, filtered through a 0.22-µm PES membrane filter, and then transferred to a 100 kDa ultrafiltration tube (Millipore). Ultrafiltration was performed by centrifugation at 3000*g*, 4°C for 10 min. To eliminate residual cryoprotectants, an equal volume of PBS was added to the ultrafiltration tubes, and the ultrafiltration process was repeated twice. The concentrated vesicle suspensions were pipetted onto carbon-coated copper grids and incubated at room temperature (RT) for 10 min. After removing excess liquid, the samples were negatively stained with 2% uranyl acetate for 1–2 min. Grids were dried naturally and observed by transmission electron microscopy (TEM; JEM-2100plus, JEOL, Japan) to characterize the morphology and structural integrity of EVs.

### Cell metabolic activity assays

L929 murine fibroblasts (Zhejiang Baidi Biotechnology, China) and immortalized human dermal fibroblasts (HDF-SV40T; Xiamen Immocell Biotechnology, China) were used for determining the effect of CM0, CM1, and CM2 on cell metabolic activity. Cells were cultured under standard culture conditions. L929 cells were maintained in Dulbecco’s Modified Eagle Medium (DMEM)-High Glucose (#12491015, Gibco, USA) supplemented with 10% fetal bovine serum (FBS) (#A5256701, Gibco, USA) and 1% penicillin/streptomycin (#15140122, Gibco, USA). HDF-SV40T cells were cultured in MEM medium (Pricella Biotechnology, China).

Cells were seeded into 96-well plates at densities of 2 × 10^3^ (L929 cells; metabolic activity), 1 × 10^4^ cells/well (L929 cells; cell viability), 4 × 10^2^ cells/well (HDFs; metabolic activity), and 4 × 10^3^ cells/well (HDFs; cell viability). After a 24-h attachment period, culture medium was aspirated and replaced with 200 μL of fresh medium containing lyophilized CM (CM0, CM1, CM2) at serial dilutions (2–500×; prepared by rehydrating lyophilized CM mixed with complete medium). The original sample solution was prepared by one tube of freeze-dried powder rehydration with 1 mL ddH_2_O; 2× group referred to as the original medium mixed with an equal volume of complete medium and the original medium was diluted twice; 4× group meant 2× group diluted twice with complete medium. On this basis, diluting twice of 4× group was denoted as 8× group and so on. Corresponding osmotic pressures of CM2 are exhibited in Supplementary Figure S2. Controls included untreated cells (control group) and medium-only blanks.

Following incubation for 48 h, cell metabolic activity was assessed using a CCK-8 assay (# BS350B, Biosharp, China). Briefly, the culture medium was sucked out, and 100 μL fresh culture medium within 10 μL CCK-8 reagent was added to each well, the plates were incubated for 2 h (L929) and 4 h (HDF) at 37°C before absorbance measurement. These durations were optimized to achieve optical density (OD) values of 1.0 ± 0.2, ensuring detection linearity. Absorbance was measured at 450 nm using an EnSight multimode plate reader (PerkinElmer, USA). Cell viability was calculated by the following formula:


$$\text{Viability (\%)}=\left({\text{OD}}_{450,\;\text{test}}-{\;\text{OD}}_{450,\;\text{blank}}\right)/\left({\text{OD}}_{450,\;\text{control}}-{\text{OD}}_{450,\;\text{blank}}\right)\times 100\%$$

where OD_450_ represents the optical density at 450 nm.

### Evaluation of cell migration via scratch wound and transwell assays

The migratory capacity of HDF in response to lyophilized conditioned medium (CM0, CM1, CM2) was assessed using complementary scratch wound healing and transwell migration assays.

#### Scratch wound healing assay

HDF-SV40T cells were seeded into 6-well plates (1 × 10^6^ cells/well) and cultured to >90% confluence. A uniform linear scratch was introduced into monolayers using a sterile pipette tip, followed by three washes with PBS buffer to remove debris. Cells were treated with relevant groups reconstituted in basal medium. The experimental design included the following groups: a negative control (basal medium only), a cryoprotectant control (trehalose combined with PVP40 at the equivalent concentration of CM2), a positive control (10% fetal bovine serum, FBS), and three experimental groups (CM0, CM1, and CM2) at the concentration of 4×.

Phase-contrast images (Nikon Eclipse Ti2, Japan) were captured at 0, 24, and 48 h posttreatment. Residual wound area was quantified as a percentage of initial area using ImageJ software (National Institutes of Health, USA). The percentage of wound closure was calculated according to the following formula:


Wound closure rate (%)=[(Initial area–Residual area)/Initial area]×100%.

#### Transwell migration assay

Transwell chambers (#3378, Corning, USA) were placed in a 24-well plate, and 100 μL 4 × 10^4^ HDF-SV40T cells/100 μL serum-free MEM were seeded into the upper chamber. The lower chamber contained 600 μL of test media, which included: serum-free MEM (negative control), MEM containing one of the lyophilized conditioned medium variants (CM1and CM2 at the concentrations of 4×, 8×, and 16×). Following 24 h incubation at standard culture conditions, noninfiltrated cells were removed by PBS washing. Cells that migrated to the lower side of the membrane were fixed with 4% paraformaldehyde (30 min), stained with 0.1% crystal violet (15 min), and imaged at 20× magnification. Infiltrated cells were quantified via automated thresholding (ImageJ) across three fields of view per membrane.

### Assessment of anti-inflammatory properties in LPS-stimulated macrophages

Lipopolysaccharide (LPS) (#ST1470, Beyotime Biotechnology, China) was used to induce inflammatory responses in RAW 264.7 macrophages (Pricella Biotechnology, China) to evaluate the anti-inflammatory activity of lyophilized CMs. Cells were cultured in DMEM (#11965092, Gibco, USA) supplemented with 10% heat-inactivated FBS (#A5256701, Gibco, USA) and 1% penicillin/streptomycin (#15140122, Gibco, USA) under standard conditions (37°C, 5% CO_2_, humidified atmosphere) and passaged every 2–3 days using a cell scraper. For experimental use, cells in logarithmic growth phase were seeded into 12-well plates at a density of 1 × 10^5^ cells/well in 1 mL complete growth medium and allowed to adhere for 12 h prior to treatment. Experiments were performed with the following groups: (i) blank group: untreated cells in complete medium; (ii) LPS-stimulated control: 100 ng/mL LPS; (iii) dexamethasone (Dex, #D4902, Sigma-Aldrich, USA)-treated control (positive control): 100 ng/mL LPS + 5 μg/mL dexamethasone; (iv) LPS + Protect (trehalose combined with PVP40 at the concentration equal to 4×); (v) LPS + CM0: 100 ng/mL LPS + CM0 (4×, 8×, 16×); (vi) LPS + CM1: 100 ng/mL LPS + CM1 (4×, 8×, 16×); (vii) LPS + CM2: 100 ng/mL LPS + CM2 (4×, 8×, 16×). Plates were incubated for 24 h at standard culture conditions, after which the supernatants were collected.

Pro-inflammatory cytokines (IL-1β, IL-6, TNF-α) were quantified by ELISA kit according to the manufacturer’s instructions. Reagent manufacturers and catalog numbers are detailed in Supplementary Table S2. Absorbance was measured at 450 nm in a plate reader (EnSight; PerkinElmer) and cytokine concentrations were derived from the standard curves. Data were normalized to the mean of untreated controls.

### Western blot analysis

Western blotting (WB) was performed to detect the expression of CD63 as well as ECM remodeling-related proteins. All experimental operations followed standard laboratory protocols.

For the detection of CD63 in lyophilized MSC-CM (Lyo-MSC-CM), sample preconcentration was conducted prior to protein extraction. Briefly, each Lyo-MSC-CM sample (CM0, CM1, and CM2) was reconstituted with 1 mL of double-distilled water, vortexed for 1 min, and filtered through a 0.22-μm PES membrane. The filtrate was transferred into a 100 kDa ultrafiltration tube (Millipore) and centrifuged at 3000*g* for 10 min at 4°C. An equal volume of PBS was supplemented, and the ultrafiltration procedure was repeated twice. The retained fraction was collected into a 1.5-mL centrifuge tube, and protein concentration was quantified using a BCA protein assay kit. All samples were normalized to a uniform concentration of 1 μg/μL, mixed with 5× protein loading buffer, and denatured at 95°C for 5 min in a metal bath. A total of 10-μg denatured protein per lane was separated by 10% SDS-PAGE, with an initial voltage of 80 V for 30 min followed by 120 V for 1 h. Separated proteins were then electrotransferred onto PVDF membranes (#IPVH00010, Millipore, USA) at 200 mA for 70 min. After blocking with 5% nonfat milk diluted in TBST for 1 h at RT, the membranes were incubated with primary antibody against CD63 (1:1000; #52090, CST, USA) for 1 h at RT. After washing with TBST for three times, membranes were incubated with HRP-conjugated goat antirabbit IgG secondary antibody (1:5000; #111-035-003, Jackson ImmunoResearch, USA) for another 1 h at RT.

For the detection of ECM-related proteins, cellular total protein was extracted using RIPA lysis buffer (#P0013B, Beyotime Biotechnology, China) containing protease and phosphatase inhibitor cocktails (#P1045, Beyotime Biotechnology, China). Protein concentration was determined via BCA assay (#P0011, Beyotime Biotechnology, China) calibrated with a BSA standard curve. A total of 20 μg denatured protein per lane was subjected to 10% SDS-PAGE and transferred to PVDF membranes using the same electrotransfer conditions described above. Membranes were blocked with 5% nonfat milk in TBST for 1 h at RT, and then incubated overnight at 4°C with primary antibodies targeting collagen type I (1:5000; #ab138492, Abcam, UK), fibronectin (FN) (1:5000; #A12977, Abclonal, China), elastin (1:500; #A22733, Abclonal, China), with GAPDH (1:10 000; #ab181602, Abcam, UK) used as the internal loading control. On the next day, membranes were rinsed with TBST and incubated with the same HRP-conjugated secondary antibody (1:5000) for 1 h at RT.

All protein bands were visualized using an ECL chemiluminescence system (UVP ChemiDoc-It 515, USA), and band intensity was quantitatively analyzed using ImageJ software. The relative expression levels of ECM related proteins were normalized to GAPDH.

### *In vivo* wound healing model

Thirty Sprague-Dawley rats (6-weeks-old, female, 200–220 g) were used to evaluate the therapeutic efficacy of lyophilized CM1 and CM2 in a full-thickness cutaneous wound repair model. All experimental protocols were conducted in compliance with institutional guidelines for animal welfare and approved by the Animal Ethics Committee of Wuhan Halic Biotechnology Co., Ltd (Approval No. HLK-20241224-003).

Under aseptic conditions and 5% isoflurane anesthesia, a full-thickness excisional wound with a diameter of 10 mm was surgically generated on the dorsal midline using a sterile biopsy punch. Rats were randomized into three groups (*n* = 10/group): (i) control group, daily topical administration of 100 μL PBS; (ii) CM1 group, treated with 100 μL reconstituted lyophilized CM1 (1 mg/mL in sterile PBS); and (iii) CM2 group, treated with 100 μL reconstituted lyophilized CM2 (1 mg/mL in sterile PBS). Treatments were applied topically to the wound bed once daily for 14 consecutive days. All wounds remained uncovered throughout the experimental period to facilitate uniform topical administration and accurate efficacy evaluation.

Wound closure kinetics were monitored via standardized digital photography (Nikon D7200) on days 0, 4, 7, 10, and 14 postsurgery. The residual wound area was calculated and normalized to the initial wound area to determine the relative wound closure rate using ImageJ software.

To further explore the potential mechanisms driving the wound healing, cytokine profiles (ELISA), histopathological evaluation, and immunohistochemical analysis were performed on different days. The sample size for each specific analysis was clearly defined as follows: wound closure rate analysis (*n* = 4 rats/group), day 7 ELISA and immunofluorescence (*n* = 3 rats/group), day 10 HE and Masson staining (*n* = 3 rats/group), and day 14 ELISA and immunofluorescence (*n* = 4 rats/group). All sample size allocations were designed to meet the statistical requirements for scientific rigor and reproducibility.

#### Cytokine profiling (ELISA)

Wound tissue samples were harvested from euthanized rats on day 7 (*n* = 3 rats/group) and 14 (*n* = 4 per group) were snap-frozen in liquid nitrogen and homogenized in ice-cold PBS (pH 7.4) containing 1:10 w/v protease inhibitors (#P8340, Sigma-Aldrich, USA). Homogenates were centrifuged (10 000*g*, 15 min, 4°C) and the resulting supernatants were collected. The expression levels of interleukin 10 (IL-10), fibroblast growth factor (FGF), and vascular endothelial growth factor (VEGF) were quantified using ELISA kit according to the manufacturer’s instructions. Reagent manufacturers and catalog numbers are detailed in Supplementary Table S3.

#### Histopathological staining

Hematoxylin and eosin (H&E) and Masson’s trichrome staining were performed on biopsies of 10-day-old wounds. Three rats per group were euthanized by cervical dislocation and full-thickness wound tissue including a 2-mm peripheral margin was excised using a sterile punch biopsy. Longitudinal sections through the wound were fixed in 4% paraformaldehyde (24 h, 4°C), dehydrated in graded ethanol–water series, embedded in paraffin, and sectioned at 4-μm thickness. For H&E staining, the paraffin-embedded sections were dewaxed in xylene, rehydrated in graded steps of water/ethanol, and stained with H&E (#C0105, Beyotime Biotechnology, China) according to standard protocol. Epidermal thickness was quantified at 100× magnification (Nikon Eclipse Ni-U, Japan) using ImageJ software. Masson’s trichrome staining (#C0189, Beyotime Biotechnology, China) was performed to evaluate collagen deposition according to the instrument. After deparaffinization and rehydration, nuclear staining, cytoplasmic and muscle fiber staining, collagen differentiation, and collagen staining were applied. Blue-stained collagen fibers were quantified as a percentage of total wound area by ImageJ.

#### Immunohistochemistry

Wound tissues were harvested and processed into paraffin sections as described above. Paraffin-embedded sections were placed on glass slides and dried in an incubator at 55°C for 30 min, subjected to antigen retrieval in 0.01 M citrate buffer (pH 6.0), blocked with 5% bovine serum albumin (1 h, RT), and incubated overnight at 4°C with primary antibodies: anti-CD31 (1:500) (#A19014, Abclonal, China), anti-IL-6 (1:100) (#A21264, Abclonal, China), and anti-TNF-α (1:100) (#ab205587, Abcam, UK). After washing with PBS, sections were incubated with Cy3-conjugated goat antirabbit IgG (1:200) (#AS007, Abclonal, China) for 1 h at RT and counterstained with 4′,6‑diamidino‑2‑phenylindole (DAPI) (#RM02978, Abclonal, China). Slides were scanned using laser scanning confocal microscopy at 40× magnification (Nikon N-STORM, Japan). The mean fluorescence intensity was quantified using ImageJ software by normalizing the integrated density to the total number of cells, as determined by DAPI-based nuclear counting.

### Statistical analysis

Unless otherwise mentioned, GraphPad Prism 9.0.0 software was utilized for data analysis and statistical evaluation. We employed one-way and two-way ANOVA followed by Tukey’s post hoc test for statistical comparisons. *P*< 0.05 was considered as statistically significant.

## Results

### Physical and chemical properties of the hUC-MSC-CM

**Figure 1:**
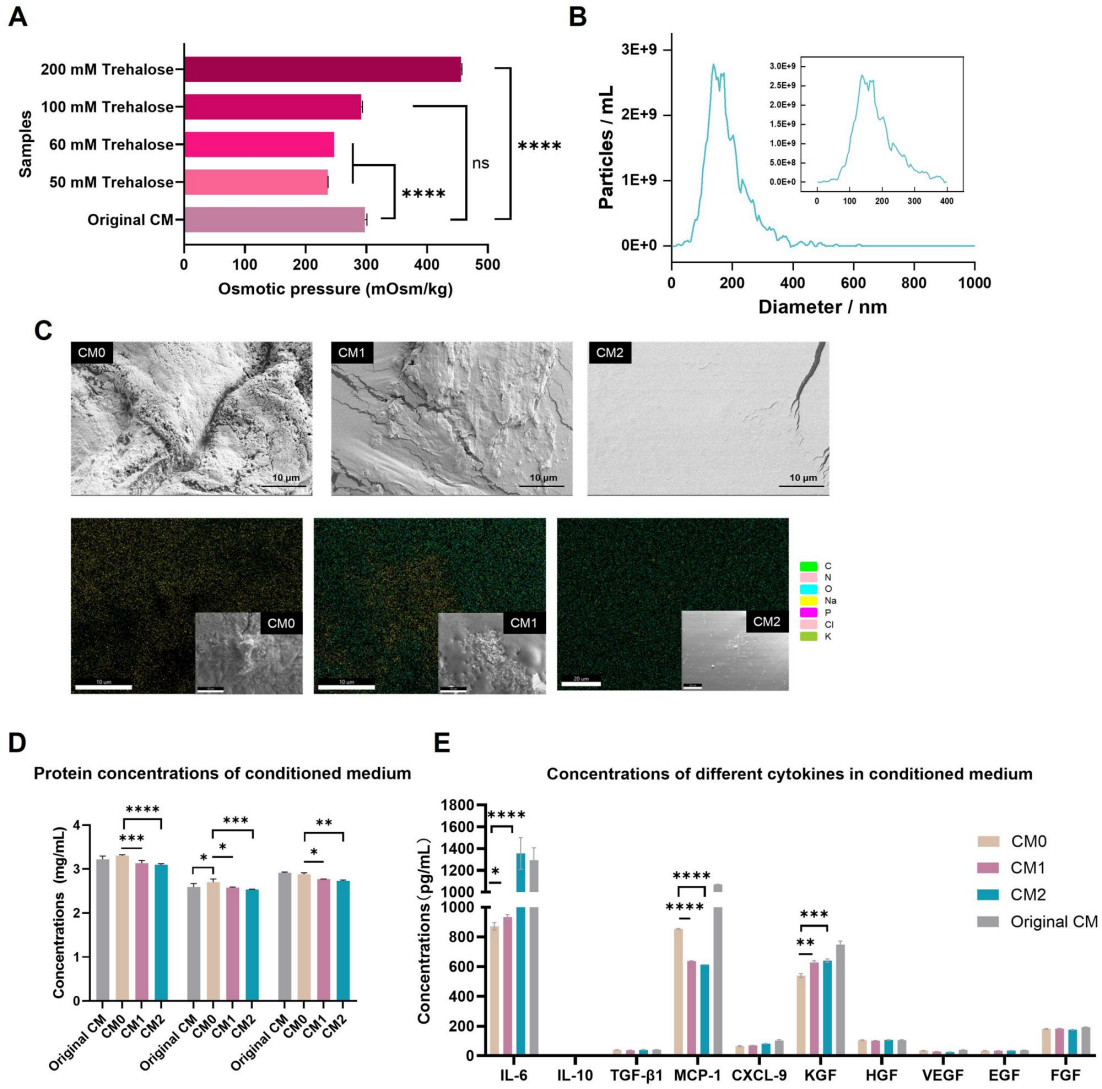
Physical and chemical properties of different treated MSC-CM. (**A**) Osmotic pressure of different concentrations of trehalose compared to the original conditioned medium, *n* = 3 per group. (**B**) a typical NTA result of CM2. (**C**) SEM photographs of different lyophilized CM and their corresponding EDS color-coded elemental mapping. (**D**) Protein content analysis by BCA kits in three batches of conditioned medium, *n* = 3 per group. Data are presented as the mean ± SEM. (**E**) Quantitative analysis of different cytokines within the four processed conditioned medium by ELISA detection method, *n* = 3 per group. Data are presented as the mean ± SEM. ns, non-significance, *P < 0.05, **P < 0.01, ***P < 0.001, ****P < 0.0001.

To systematically evaluate the role of cryoprotectants, three lyophilized formulations were compared: CM0 (unprotected MSC-CM), CM1 (trehalose-stabilized MSC-CM), and CM2 (dual cryoprotectant MSC-CM with trehalose and PVP40). First, osmotic pressure was determined to screen the optimal trehalose concentration for formulation construction. It was confirmed that 100 mM trehalose dissolved in PBS/ddH_2_O mixed buffer has the similar osmotic pressure to the original conditioned medium (Figure 1A, Supplementary Figure S2). Accordingly, 100 mM trehalose was selected as the basic cryoprotectant for subsequent lyophilization to maintain the physiological osmotic microenvironment of bioactive components. On this basis, CM1 was defined as MSC-CM lyophilized with 100 mM trehalose, CM2 as the combined formulation of 100 mM trehalose plus 2% PVP40, and CM0 as the blank lyophilized control without any protectant addition.

Subsequently, SEM combined with EDS was performed to characterize the macroscopic morphological and elemental distribution features of lyophilized powders (Figure 1C, Supplementary Figure S3). Obvious morphological differences were observed among the three groups. CM0 displayed abundant granular agglomeration and rough surface morphology. By comparison, CM1 presented a relatively flat surface with only sporadic particle aggregation regions, whereas CM2 exhibited an even and completely smooth microstructure. EDS elemental analysis further confirmed that sodium and chlorine elements dominated the surface of CM0, indicating severe salt crystallization and agglomeration during lyophilization. Meanwhile, carbon element was predominantly distributed in CM1 and CM2, suggesting that cryoprotectant molecules formed a uniform protective matrix on the sample surface. Particularly in CM2, the synergistic effect of trehalose and PVP40 enabled homogeneous distribution of protectants and contributed to the formation of a compact and smooth lyophilized structure.

Nanoparticle tracking analysis (NTA) was further applied to characterize the concentration and size distribution of EVs in rehydrated lyophilized samples. A representative NTA profile of CM2 showed that particle size was mainly distributed in the range of 100–200 nm, with a baseline concentration of 6.9 × 10^10^ particles/mL (Figure 1B). NTA results of Original CM, CM0, and CM1 are provided in Supplementary Figure S4, with particle concentrations of 1.4 × 10^10^, 8.1 × 10^9^, and 6.1 × 10^10^ mL^−1^, respectively. Notably, both CM1 and CM2 presented significantly higher nanoparticle abundance than CM0 and the original fresh CM, whereas CM0 showed an obvious reduction in particle number relative to Original CM. In terms of particle size, no substantial difference was observed between CM0 and Original CM, while CM1 and CM2 exhibited a slight increase in particle diameter. This phenomenon may be explained by hydrogen bond formation between cryoprotectant molecules and EV surface biomolecules, accompanied by coencapsulation of surrounding cytokines and soluble proteins, thereby slightly increasing the overall particle size.

To elucidate the underlying reason of the distinct NTA results among groups, a BCA assay was first conducted to quantify the total protein content of all samples (Figure 1D). Unexpectedly, CM0 possessed a markedly higher total protein level than CM1 and CM2, which seemed contradictory to its lower EV particle abundance detected by NTA. Considering that the BCA assay cannot differentiate free soluble proteins from intravesicular proteins, we further performed WB to detect the expression of the classic EV surface marker CD63 to verify the integrity and retention of EVs in each group (Supplementary Figure S5A). Quantitative grayscale analysis of WB results demonstrated that the relative CD63 expression levels of CM1 and CM2 were 1.67-fold and 1.53-fold higher than that of CM0, respectively, collectively supporting the superior retention of intact EVs in these two groups. Further TEM observation was conducted to visualize the ultrastructure of rehydrated EVs. Typical TEM image of CM2 retained the characteristic membrane morphology of EVs, despite unavoidable background protein impurities caused by direct rehydration without additional purification (Supplementary Figure S5B).

Combined with the analysis of NTA results, protein quantification and EV marker expression, the divergent results of different groups could be clarified. The elevated total protein content in CM0 was primarily attributed to EV rupture and intravesicular protein leakage during lyophilization, resulting in massive free protein release and a higher soluble protein abundance, while its intact EV number was greatly reduced. In contrast, CM1 and CM2 exerted prominent protective effects on EV structure, which effectively prevented vesicle rupture and protein leakage during lyophilization. Compared to Original CM, CM1, and CM2 showed no increase in total protein content, therefore, the increased nanoparticle counts in CM1 and CM2 in NTA results were most likely attributed to improved preservation of intact EVs combined with soluble protein assemblies stabilized by cryoprotectants that exhibit similar size ranges as EVs; however, this interpretation remained to be directly validated.

To evaluate the retention of bioactive soluble factors, ELISA was performed to quantify the levels of multiple key cytokines in Original CM, CM0, CM1, and CM2 (Figure 1E). Among the detected factors, KGF was the predominant pro-regenerative mediator among the five growth factors, with concentrations above 600 pg/mL, while IL-6 and monocyte chemoattractant protein-1 (MCP-1) dominated inflammatory regulatory factors, implying that MSC-CM possessed inherent potential in modulating wound inflammatory microenvironment. Notably, certain cytokines exhibited divergent concentration trends when co-lyophilization with cryoprotectants: levels of IL-6 and KGF increased, whereas MCP-1 decreased. These distinct alterations indicated that cryoprotectants exerted selective protective effects rather than uniform stabilization on all cytokines, which may be potentially attributable to differential binding affinities between specific cytokines and the cryoprotectant matrix. The biological functions of all detected cytokines are summarized in Supplementary Table S4. Overall, although minor changes were observed in individual factor levels, most bioactive components in lyophilized groups were well maintained comparable to fresh Original CM, confirming that the optimized lyophilization procedure did not cause massive loss of soluble bioactive molecules.

For batch-to-batch reproducibility, multibatch validation of NTA and total protein content was conducted. The total protein concentration across independent batches fluctuated within a narrow range of 2.5–3.5 mg/mL (Supplementary Figure S6), and the working concentration adopted for subsequent *in vitro* and *in vivo* experiments was unified at 3.0 mg/mL. Five independent batches of CM2 maintained stable nanoparticle concentrations at the order of 10^10^ particles/mL, with peak diameters consistently distributed at 100–150 nm (Supplementary Figure S7), demonstrating good production reproducibility of the CM2 formulation.

Finally, long-term storage stability of CM2 was evaluated by detecting characteristic cytokine levels before and after one-year storage at −20°C (Supplementary Figure S8). Representative cytokines including TGF-β1, MCP-1, and KGF were selected to cover low, medium, and high concentration gradients. No significant differences in cytokine abundance were observed between freshly prepared and one-year-stored CM2 samples. These collective results confirmed that the CM2 lyophilized formulation possessed excellent batch reproducibility and long-term storage stability, and could sustainably maintain the structural integrity and biological activity of MSC-CM during long-term preservation.

### *In vitro* effects of Lyo-MSC-CM on cell metabolic activity

To evaluate the bioactivity of lyophilized MSC-CM formulations (CM0, CM1, CM2), dose-dependent effects on cellular metabolic activity were first assessed in murine (L929) fibroblasts first via CCK-8 assay. The experimental groups were set based on volumetric dilution gradients of reconstituted lyophilized CM (details could be found in the “Transmission Electron Microscopy” section), and each dilution was further calibrated and standardized by absolute total protein concentration. The unified baseline protein concentration of undiluted reconstituted CM was 3.0 mg/mL. Accordingly, the final protein concentration was 1.5 mg/mL for the 2× group, 0.75 mg/mL for the 4× group, and serially diluted proportionally for subsequent lower concentration groups. For concise description in the manuscript, dilution multiples are adopted for unified labeling.

**Figure 2:**
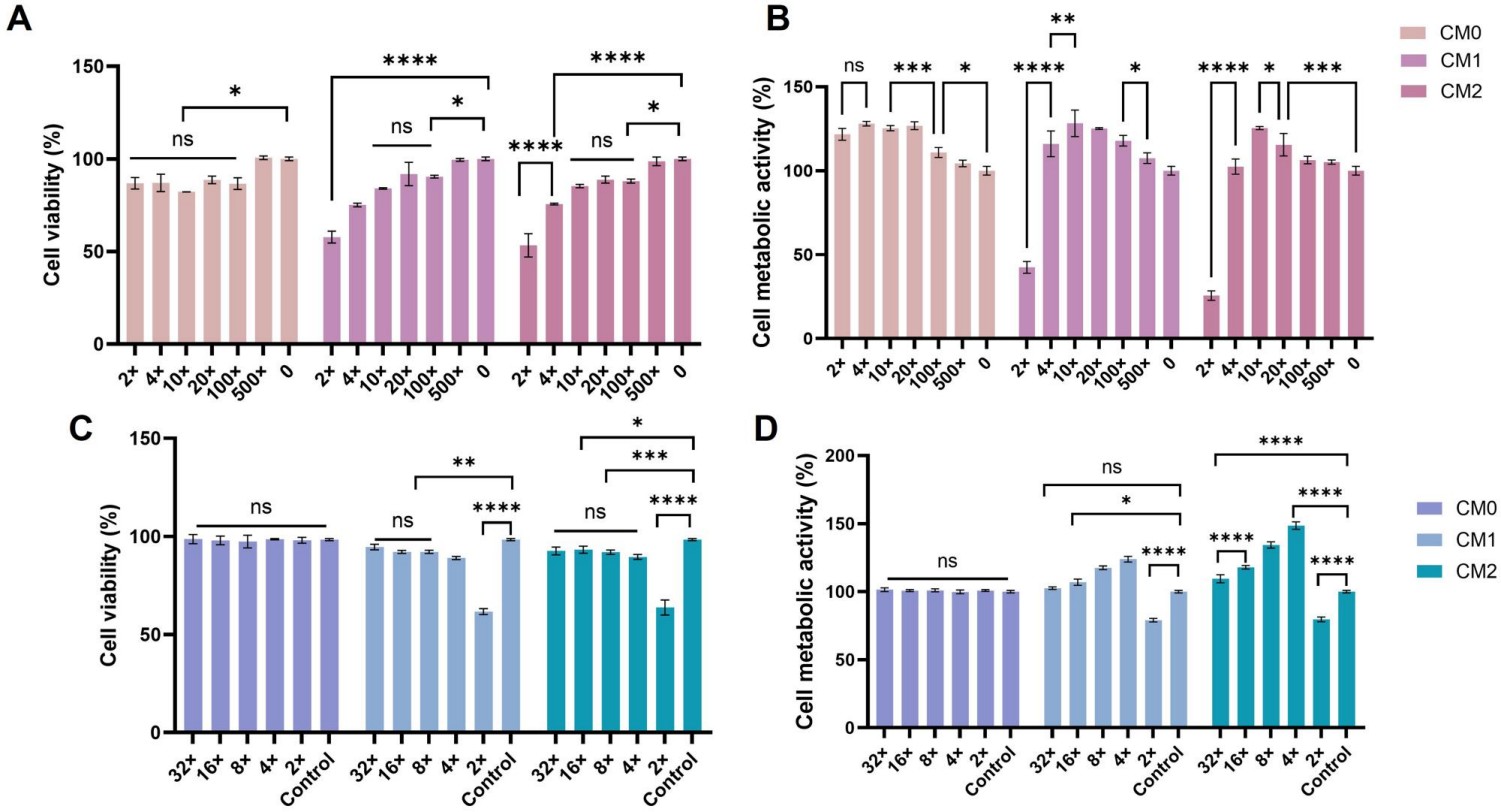
Effect of lyophilized conditioned mediums on cell viability with CCK-8 kit. (**A**, **B**) Effects on L929 fibroblast cell line. (**C**, **D**) Effects on human dermal fibroblasts (HDF). (**A**) The cell viability was measured after incubation of the cells with increasing concentrations of conditioned medium for 48 h. Corresponding seeding density was 10 000/well. (**B**) The cell metabolic activity was measured as the same way as cell viability but with a seeding density of 2000/well. (**C**) The cell viability detection of HDF with a seeding density of 4000/well. (**D**) Status of cell metabolic activity on HDF detected with a seeding density of 400/well. Data are presented as the mean ± SEM performed in triplicate. ns, nonsignificance, *P < 0.05, **P < 0.01, ***P < 0.001, ****P < 0.0001.

In L929 cells, CM0 exhibited no cytotoxicity at any concentration, while CM1 and CM2 demonstrated minimal cytotoxicity (∼85% viability) at 2× (Figure 2A). All formulations improved cellular metabolic activity within specific dose ranges (Figure 2B): CM0 enhanced metabolic viability at higher concentrations (>4×), whereas CM1 and CM2 showed maximal promoting effects at lower doses (10–500×), with modest dose dependence.

While L929 murine fibroblasts provided valuable preliminary data on CM-induced cell metabolism, subsequent functional analyses utilized HDF to better reveal the human wound healing mechanisms. Since the low-concentration conditioned medium exerted negligible influence on L929 cellular metabolic activity, the concentration range was further refined in HDF (Figure 2C and D). The overall trend was similar to L929 but there were notable differences in specific areas. In HDF cellular metabolic profiles, CM2 outperformed CM1 at elevated concentrations, and the most pronounced promoting effect occurred at a dilution ratio of 4× (CM2: 1.5-fold, CM1: 1.2-fold). Notably, CM0 lacked significant improvement in HDF cell viability (*P *> 0.05). It was aligned with prior reports that umbilical cord- and adipose-derived MSC-CM did not alter HDF viability [43, 44], but in contrast with a few studies which reported UC-MSC-CM-driven HDF proliferation [45]. The specific mechanism remained unknown, we assumed this discrepancy potentially attributable to interdonor MSC heterogeneity, culture condition variations (especially the differences of cell culture media), or lyophilization-induced attenuation of labile factors. Crucially, compared to CM0, both CM1 and CM2 retained potent bioactivity and markedly enhanced cellular metabolic viability at all tested dilution ratios ranging from 4× to 16×. Notably, CM2 exhibited superior biological efficacy relative to CM1 across the entire concentration range (Supplementary Figure S9). Moreover, control experiments confirmed that trehalose and PVP40 alone exerted no significant promotive effects on cellular metabolism at the corresponding concentrations, excluding the potential interference of cryoprotectants on cell viability (Supplementary Figure S10).

However, it should be noticed that CM1 and CM2 exhibited cytotoxicity at the dilution factor of 2×. This adverse phenomenon could be explained by two potential reasons. First, previous literature has claimed that excessively high trehalose concentrations could suppress fibroblast activation, inhibit myofibroblast transformation, and induce a senescence-like phenotype in fibroblasts, thereby impairing cell proliferation [46, 47]. Second, the high concentration (2×) of CM2 slightly elevated the osmotic pressure beyond the physiological range (340 mOsm/kg), generating an unfavorable hyperosmotic microenvironment that restricted fibroblast growth, while other concentrations were maintained within physiological osmolarity (290–310 mOsm/kg) (Supplementary Figure S2D).

These results indicated that appropriate concentrations of cryoprotectants effectively preserved the bioactivity of MSC-CM during lyophilization and facilitated fibroblast metabolic activity, which could support cellular growth in the context of wound repair.

### Lyo-MSC-CM enhances fibroblast migration *in vitro*

To assess the role of lyophilized MSC-CM (Lyo-MSC-CM) in wound repair, scratch wound healing and transwell migration assays were performed on HDFs treated with optimized dilution ratios (16×, 8×, 4×).

**Figure 3:**
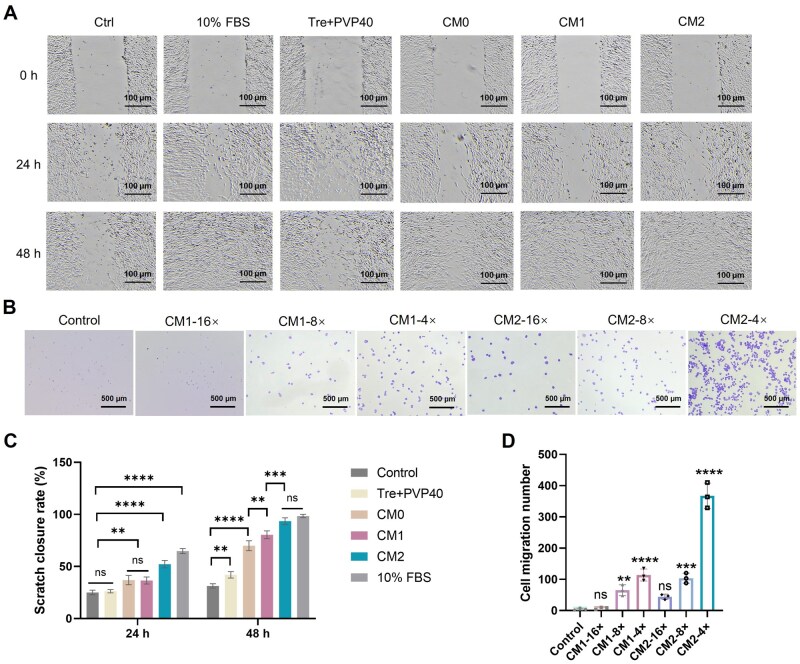
Effect of lyophilized conditioned mediums on cell migration by scratch and transwell assays. (**A**) Photos of wound healing assays after scratching for 0, 24 and 48 h, scar bar: 100 µm. (**B**) Representative microscopic images of cells that migrated to the upper transwell in transwell migration assay, scale bar: 500 µm. (**C**) Scratch closure rate of (A), *n* = 3 per group. (**D**) The quantitation analysis of (**B**), *n* = 3 per group. Data are presented as mean ± SEM. Significant difference analysis referred to the comparison between the experimental groups and the control group. ns, nonsignificance, **P < 0.01, ***P < 0.001, ****P < 0.0001.

Cell scratch assays were conducted to evaluate the migratory capacity of fibroblasts under different treatments (Figure 3A and C). The pure cryoprotectant treatment exerted a negligible effect on cell migration at 24 h and only induced a slight promotion of cell motility at 48 h, indicating that the cryoprotectant mixture itself had a minimal auxiliary effect on fibroblast migration. In contrast, all lyophilized MSC-CM groups significantly facilitated scratch wound closure. At 24 h, the wound closure rates of CM0, CM1, and CM2 (4×) were 37.0%, 36.6%, and 52.2%, respectively, all of which were markedly higher than that of the PBS control group (25.1%). Notably, the high-concentration CM2 group achieved a wound healing effect comparable to the 10% FBS positive control at 48 h, with a nearly 100% scratch wound closure rate.

Transwell migration assays further corroborated the scratch wound findings (Figure 3B and D). Both the 8× and 4× dilution ratios of CM2 induced a pronounced increase in the number of migrated cells compared to the negative control (*P *< 0.01), with the 4× CM2 group showing the highest migratory activity. Collectively, these results indicated that cryoprotectants alone exerted only a minor stimulatory effect, while cryoprotectant-stabilized MSC-CM significantly promoted fibroblast motility in a concentration-dependent manner. The superior efficacy of CM2 suggested that the optimized lyophilization formulation effectively preserved key bioactive factors, supporting the therapeutic potential of Lyo-MSC-CM to facilitate fibroblast recruitment during cutaneous wound healing.

### Anti-inflammatory effect of Lyo-MSC-CM *in vitro*

**Figure 4:**
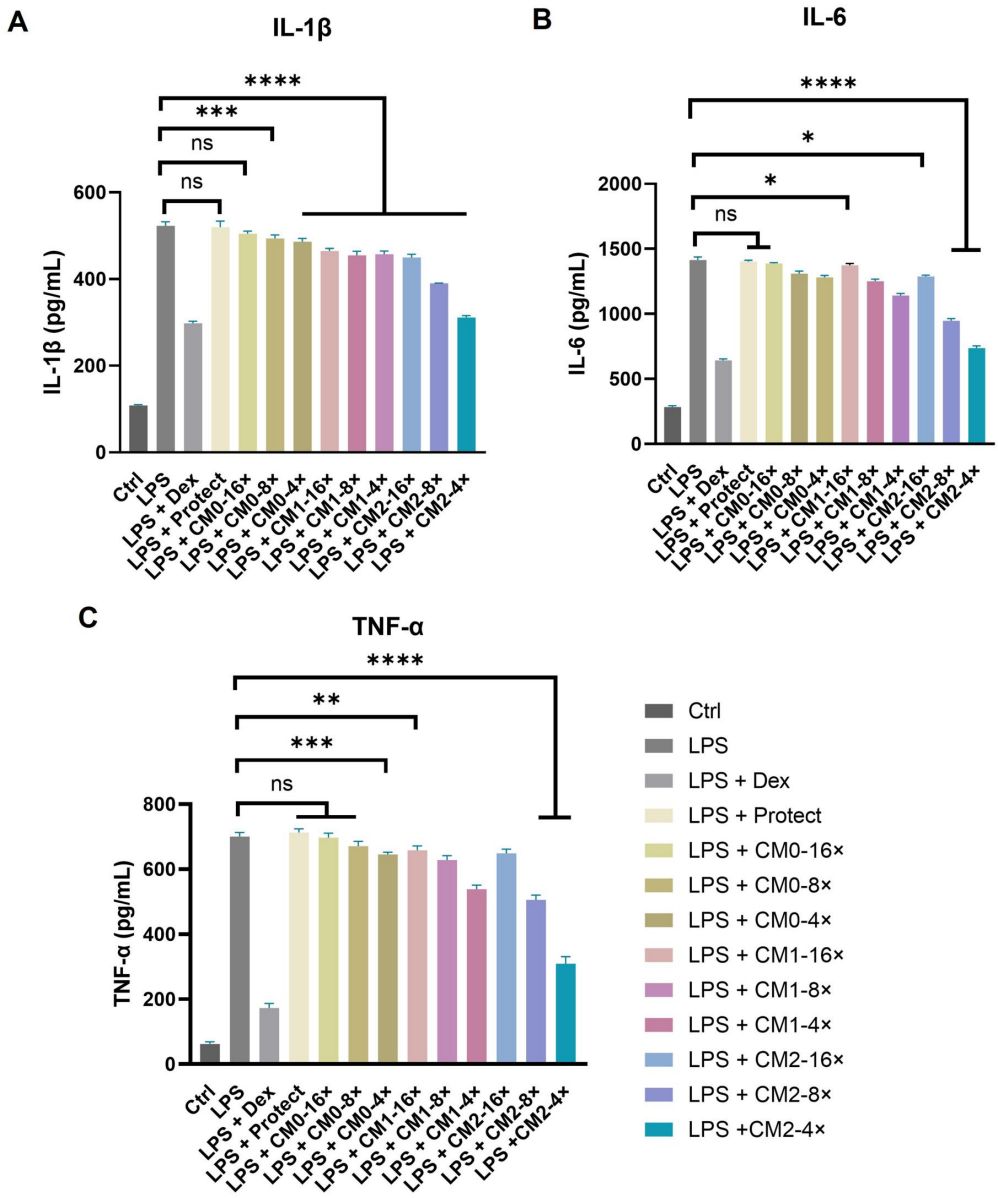
Lyo-MSC-CM regulated pro-inflammatory cytokine secretion *in vitro*. (**A–C**) Quantification of IL-1β (**A**), IL-6 (**B**), and TNF-α (**C**) secretion levels across experimental groups by ELISA. Data are represented as mean ± SEM of *n* = 3 independent biological replicates. ns, nonsignificance, *P < 0.05, **P < 0.01, ***P < 0.001, ****P < 0.0001.

The anti-inflammatory activity of Lyo-MSC-CM was assessed in LPS-stimulated RAW264.7 macrophages via quantification of IL-1β, IL-6, and TNF-α secretion (Figure 4). As verified by the results, the blank cryoprotectant group without MSC-CM exhibited no observable anti-inflammatory effect, confirming that the pure protective agent itself cannot alleviate LPS-induced inflammatory responses. For lyophilized sample groups, CM0 without optimized cryoprotectant modification retained a certain degree of intrinsic anti-inflammatory activity of MSC-CM and could slightly reduce pro-inflammatory cytokine secretion; however, its immunomodulatory efficacy was extremely limited and significantly weaker than that of cryoprotectant-modified CM1 and CM2. High-concentration formulations (8× and 4×) of both CM1 and CM2 significantly suppressed pro-inflammatory cytokine release compared to LPS-treated controls (*P *< 0.05), and CM2-4× exhibited the most potent inhibition, reducing IL-1β, IL-6, and TNF-α levels by 40.5%, 47.9%, and 55.9%, respectively (*P *< 0.0001 compared to LPS group). Furthermore, the anti-inflammatory effect of CM2 exhibited a dose-dependent manner, with CM2-4× demonstrating comparable efficacy to dexamethasone (positive control). This indicated that cryoprotectant-optimized MSC-CM retains its immunomodulatory properties, and effectively mitigated LPS-induced inflammation.

### MSC-CM modulated ECM synthesis *in vitro*

**Figure 5:**
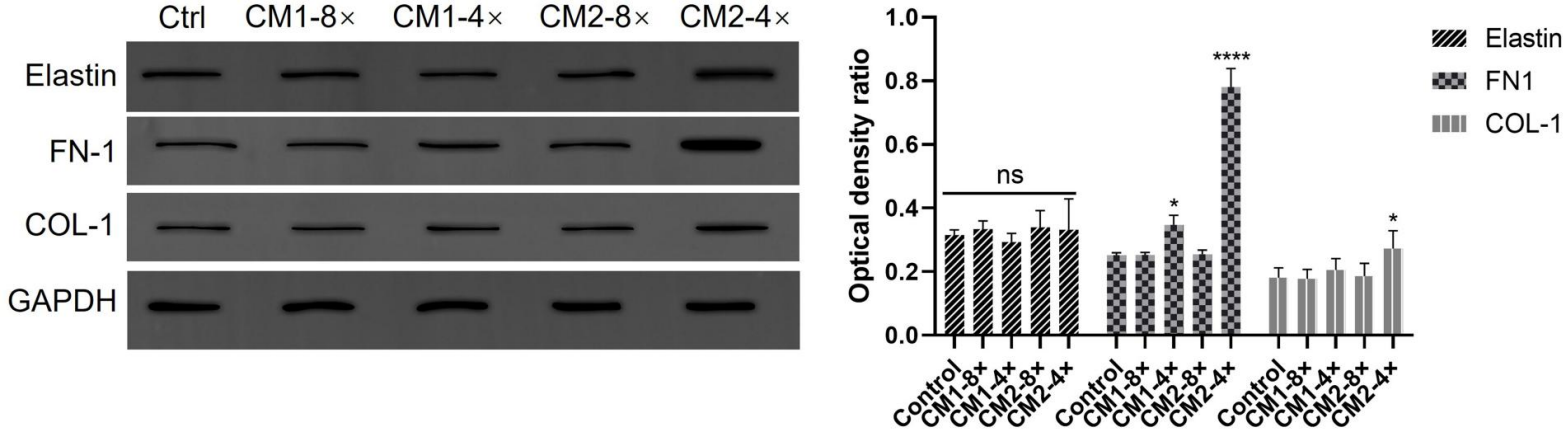
Western blot analysis of Lyo-MSC-CM on the expression of extracellular matrix. Left: the expression of elastin, FN-1, and COL-1 in the treatment of different concentrations of Lyo-MSC-CM; Right: corresponding analysis of the left, *n* = 3. Data are presented as mean ± SEM. Significant difference analysis referred to the comparison between the experimental groups and the control group. ns, nonsignificance, *P < 0.05, ****P < 0.0001.

Western blot analysis was conducted to quantify the effects of lyophilized MSC-CM (Lyo-MSC-CM) on ECM protein expression, focusing on elastin, FN-1, and type I collagen (COL-1) (Figure 5). Elastin is a critical determinant of dermal elasticity and is important in maintaining the elasticity of blood vessels. FN-1 is a kind of glycoprotein that participates in cell adhesion and migration processes, and COL-1 is one kind of fibrous collagen and is of great significance to skin, bone, and connective tissue. Based on our preceding cell functional results, only the optimal high-concentration Lyo-MSC-CM (dilution times at 4× and 8×) was selected for subsequent ECM detection. The protein quantification results demonstrated that the application of Lyo-MSC-CM had no significant effect on the expression level of elastin (*P *> 0.05), but significantly improved the expression level of FN-1 with high concentrations (CM1-4× and CM2-4×, 1.36-fold and 3.05-fold compared to control, respectively), which directly benefited wound healing. The expression level of COL-1 also increased by 1.51-fold with the treatment of CM2-4×, suggesting that CM2-4× enhanced collagen synthesis. These findings indicated that CM2 selectively orchestrated ECM remodeling by augmenting FN-1 and COL-1 synthesis, thereby supporting enhanced tissue repair kinetics in wound healing models.

### *In vivo* wound healing effects of Lyo-MSC-CM

**Figure 6:**
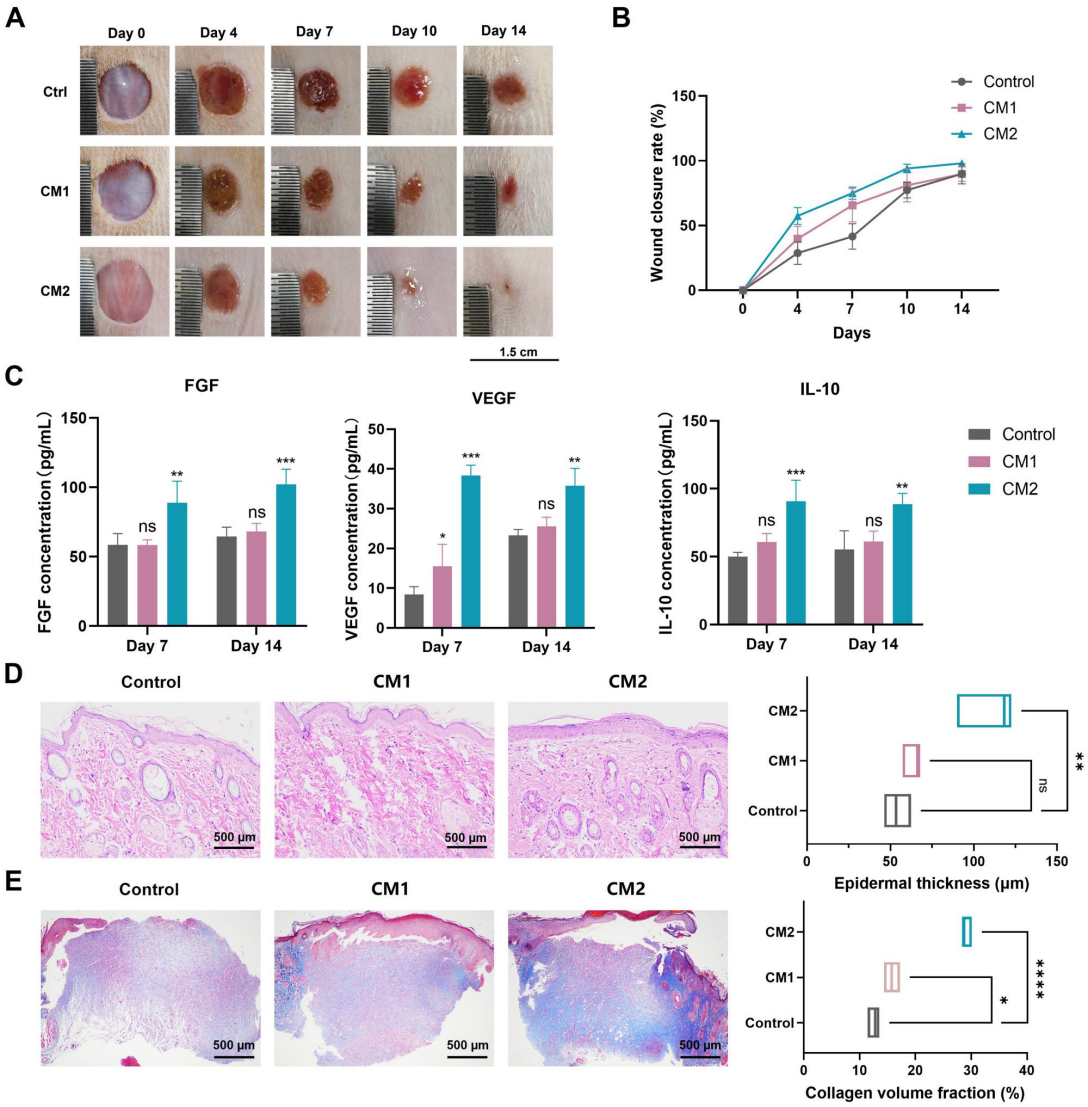
*In vivo* wound healing effects of Lyo-MSC-CM. (**A**) Representative macroscopic images of skin wound sites in rats treated with PBS, CM1 and CM2. (**B**) Quantitative analysis of wound closure rate (*n* = 4 per group). (**C**) ELISA detection of FGF, VEGF, and IL-10 expression levels (*n* = 3 per group). (**D**) H&E staining of wound tissues at day 10 and statistical analysis of epidermal thickness, *n* = 3 per group (scale bar: 500 μm). (**E**) Masson’s trichrome staining of wound tissues at day 10 and quantification of collagen volume fraction, *n* = 3 per group (scale bar: 500 μm). All quantitative data are presented as mean ± SEM. ns, non-significance difference; *P < 0.05, **P < 0.01, ***P < 0.001, ****P < 0.0001.

Rats were randomly divided into three groups: control (PBS treated), Experimental Group 1 (CM1), and Experimental Group 2 (CM2). Circular full-thickness wounds (10 mm) were created under anesthesia and digitally photographed on days 0, 4, 7, 10, and 14. Wound areas were measured using ImageJ software (Figure 6A and B, Supplementary Figure S11). It demonstrated that Lyo-MSC-CM treatment could reduce wound area compared to the control, while CM2 treatment exhibited a more significant wound healing effect, especially during the first 10 days. Topical CM2 administration significantly accelerated wound closure, especially in the first ten days (e.g., achieving a 75.0% reduction in wound area by day 7 compared to 41.6% in controls), and CM2-treated wounds exhibited near-complete epithelialization by day 14, whereas controls retained residual defects.

Quantitative ELISA on day 7 and 14 demonstrated that CM2 upregulated the expression level of pro-regenerative cytokines, including FGF (1.5-fold on day 7 and 1.6-fold on day 14) and VEGF (4.5-fold on day 7 and 1.5-fold on day 14), alongside anti-inflammatory cytokine IL-10 (1.8-fold on day 7 and 1.6-fold on day 14) (Figure 6C). Histological analysis (H&E staining) on day 10 (Figure 6D) showed an increased epidermal thickness of using CM2 compared to the control group (54.1 μm in control and 110.3 μm in CM2), indicating the ability of re-epithelialization of CM2. Moreover, Masson’s trichrome staining (Figure 6E) also demonstrated enhanced collagen deposition in CM-treated wounds (1.3-fold in CM1 and 2.3-fold in CM2 compared to control). The staining results together revealed that CM2 accelerated tissue reconstruction while enhancing ECM remodeling.

**Figure 7:**
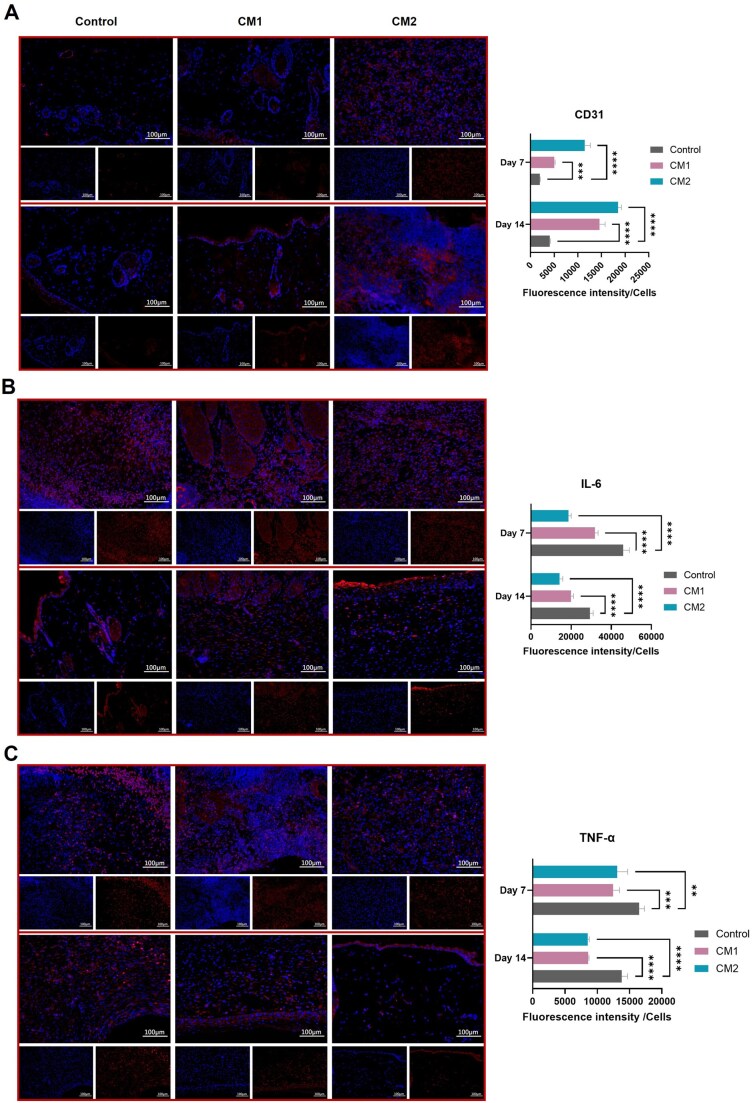
Immunofluorescence staining results in wounds. Blue color in the images represents fluorescence staining for cell nuclei with DAPI. (**A**) Images of stained wound tissue cells for CD31 (red) and corresponding analysis of fluorescence intensity. (**B**) Images of stained wound tissue cells for IL-6 (red) and corresponding analysis of fluorescence intensity. (**C**) Images of stained wound tissue cells for TNF-α (red) and corresponding analysis of fluorescence intensity. Quantitative data represent the mean ± SEM of *n* = 3/group. **P < 0.01, ***P < 0.001, ****P < 0.0001.

To further elucidate the related mechanisms, we also conducted immunofluorescence profiling of CD31, IL-6, and TNF-α (Figure 7). The results indicated that CM2 treatment upregulated pro-angiogenic markers, with CD31^+^ endothelial cell density elevated by 5.8-fold and 4.0-fold compared to PBS treatment on day 7 and day 14, respectively (Figure 7A). And it was also higher than CM1 treatment (2.3-fold and 1.2-fold compared to CM1 on day 7 and day 14, respectively). Additionally, both the pro-inflammatory cytokines IL-6 and TNF-α were significantly lowered down by CM2 treatment (*P *< 0.05) (Figure 7B and C). The capacity of CM2 to suppress pro-inflammatory cytokines was in well accordance with the previous ELISA results in LPS-stimulated macrophages. The above data collectively demonstrated that CM2 promoted wound healing through multifaceted mechanisms, including propagating angiogenesis (upregulating VEGF and CD31), driving proliferative signaling (increasing FGF to activate fibroblast), modulating immune responses (suppressing IL-6 and TNF-α, and elevating IL-10), and enabling ECM remodeling (enhancing re-epithelialization and collagen synthesis).

## Discussion

**Figure 8:**
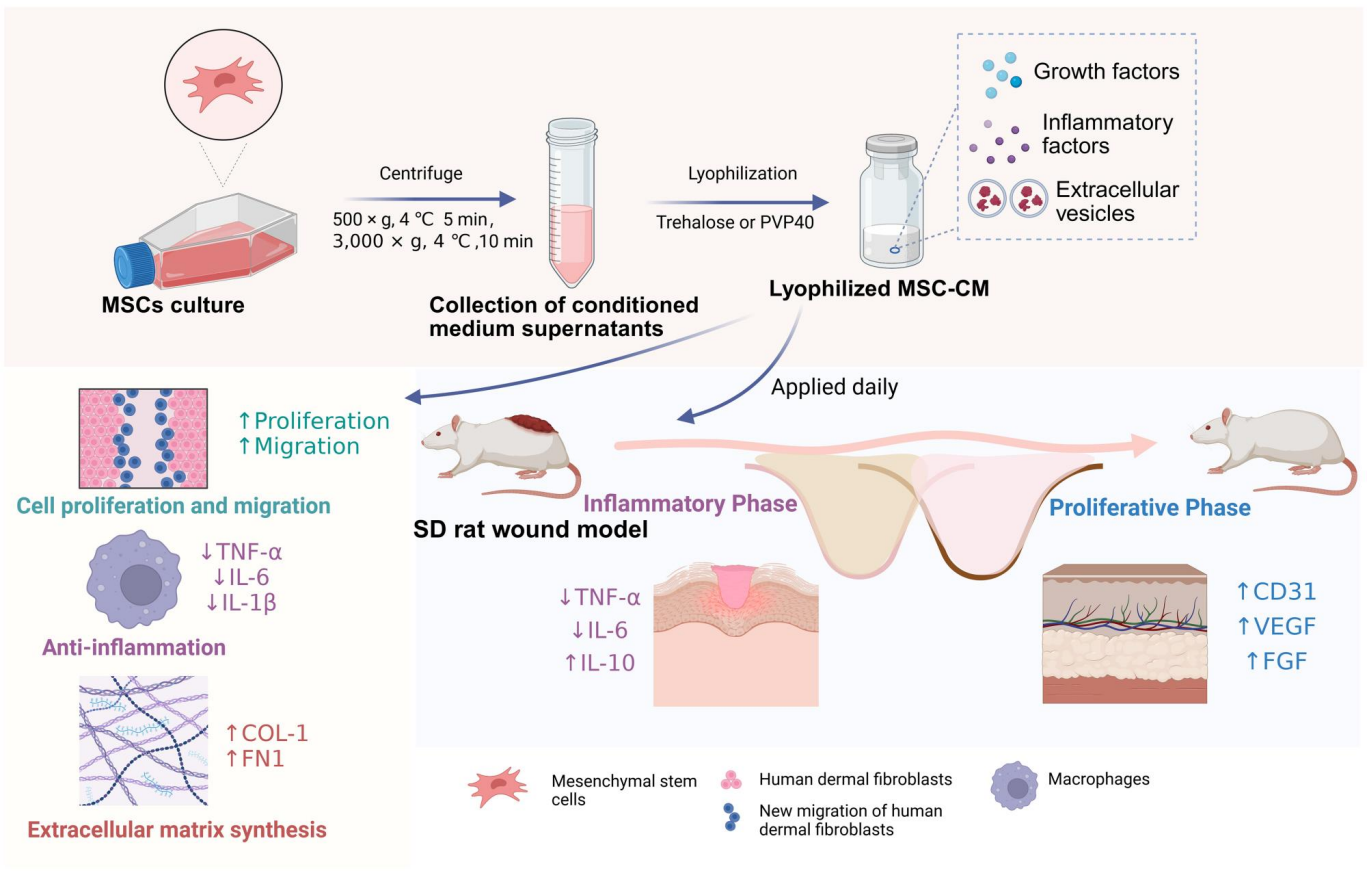
Schematic drawing of experimental procedures and results (figure was created with Biorender.com).

This study investigated the therapeutic potential of cryoprotectant-supplemented lyophilized mesenchymal stem cell conditioned medium (Lyo-MSC-CM) in promoting cutaneous wound healing through coordinated modulation of inflammatory and proliferative phases (Figure 8). The Lyo-MSC-CM contained bioactive components including growth factors (e.g., VEGF, KGF), immunomodulatory cytokines (e.g., IL-6, MCP-1), and EVs, with cryoprotectants significantly enhancing nanoparticle retention and amplifying therapeutic potency. MSC-CM lyophilized with trehalose and PVP40 (CM2) exhibited superior functional outcomes compared to unprotected formulations and only one cryoprotected formulations (CM0 and CM1). *In vitro* analyses confirmed that CM2 enhanced fibroblast proliferation and migration, attenuated macrophage release of pro-inflammatory cytokines (IL-1β, IL-6, TNF-α), and upregulated ECM synthesis, while *in vivo* evaluation in a rat full-thickness wound model revealed accelerated closure kinetics.

### Content and compositional characteristics of MSC-CM

The composition of MSC-CM exhibits significant heterogeneity across tissue sources, which directly influence its biological activity. It has been well documented that AD-MSC-CM is rich in pro-angiogenic factors including VEGF, FGF2, hepatocyte growth factor (HGF), angiopoietin-1, and pigment epithelium-derived factor (PEDF) [48], while BM-MSC-CM predominantly secretes IGF-1, TGF-β1, and HGF to participate in angiogenesis and tissue remodeling [49]. In comparison, UC-MSC-CM possesses a unique secretory profile with abundant cytokines, especially higher concentrations of proinflammatory mediators such as IL-6 and MCP-1 [50, 51]. Consistent with previous proteomic and cytokine profiling evidence, our study exhibited that UC-MSCs cultured in serum-free 2D conditions secreted IL-6, MCP-1, and KGF as principal immunomodulatory factors within the detected 10 cytokines. This secretory profile underscored the dual role of UC-MSC-CM: while MCP-1 and IL-6 modulated the inflammatory responses, KGF and other growth factors promoted cell proliferation and migration to accelerate wound healing. It was noteworthy that the present study only performed targeted quantification of representative inflammatory and reparative cytokines via ELISA. Given the high complexity of the MSC secretome, targeted detection cannot fully reflect the overall component changes of MSC-CM after lyophilization. Therefore, future proteomic studies would be valuable to comprehensively characterize the impact of lyophilization on the entire secretome profile.

### Cryoprotectants in MSC-CM

Cryoprotectants are widely utilized to alleviate freeze-drying damage to biological substances such as functional proteins, probiotics and living cells, primarily via inhibiting ice crystal formation and stabilizing macromolecular structures. At present, most relevant studies mainly focus on the application of single cryoprotectant such as trehalose or simple combined formulations in the preservation of EVs. Charoenviriyakul *et al*. first demonstrated that lyophilization of exosomes with trehalose did not alter the pharmacokinetics of exosomes and could be used to preserve exosomes at RT [39], later Bosch *et al.* reported that the addition of trehalose could narrow EV size polydispersity and enhance nanoparticle yield [52]. Susa *et al*. systematically evaluated different formulations in freeze-drying process and demonstrated that sugars in combination with dextran and glycine maintained the integrity of EVs from B lymphocytes [53]. El Baradie *et al*. claimed that lyophilization of EVs resulted in a significantly reduced number of particles, but could be attenuated by adding cryoprotectants to the EVs solution [42]. Nevertheless, relevant exploration and systematic formulation optimization are still lacking for the lyophilization preservation of complete MSC-CM, and compound cryoprotectant strategies have rarely been attempted in MSC-CM system.

In contrast to prior investigations concentrating only on exosome integrity after cryoprotection, this work comprehensively evaluated the preservative effects of trehalose and PVP40 on Lyo-MSC-CM by integrating morphological observation, nanoparticle quantification, EV biomarker identification, total protein detection, cytokine profiling, batch reproducibility verification and long-term stability assessment. SEM–EDS morphological analysis showed that CM0 without cryoprotectant presented severe salt crystallization and granular agglomeration, while CM1 and CM2 formed uniform and flat protective surfaces, indicating that trehalose and PVP40 could effectively inhibit salt precipitation and optimize the microscopic morphology of lyophilized powder. Further NTA results demonstrated that CM1 and CM2 achieved much higher nanoparticle retention than CM0, even significantly exceeding the particle concentration of Original CM. WB detection of the EV specific marker CD63 further verified the protective effect of cryoprotectants. Compared to CM0 with poor EV retention, CM1 and CM2 maintained higher CD63 protein expression, demonstrating that trehalose alone or combined with PVP40 could effectively preserve EV structural integrity. BCA total protein analysis also supported the protective mechanism of cryoprotectants, that the elevated total protein level in CM0 originated from the rupture of large amounts of EVs and the release of intravesicular proteins, and CM1 and CM2 maintained stable total protein levels which minimized vesicle breakage and internal protein leakage. Combined with WB, EV ultrastructural observation and total protein quantification, the phenomenon of the NTA results could be reasonably explained from two aspects. On the one hand, cryoprotectants effectively inhibited EV aggregation and structural disruption during freeze-drying, retaining more intact nanoparticles and thereby restoring a higher detectable EV abundance. On the other hand, the extra particle fraction exceeding the original EV concentration was most likely attributed to the soluble proteins inherent in conditioned medium, which were well protected by cryoprotectants and tended to form mild protein aggregates with a size range comparable to natural EVs. These protein aggregates were synchronously recognized and counted by NTA, and led to the higher particle concentration observed in CM1 and CM2.

ELISA cytokine profiling revealed a dichotomous response among cytokines in cryoprotectant-facilitated freeze-drying, within which IL-6 and KGF exhibited significant concentration increases, and MCP-1 demonstrated a marked reduction after lyophilization with cryoprotectants. We attributed the observed phenomena to the differing binding affinities between cryoprotectants and cytokines, as well as the selective degradation during phase transitions. Although the mechanisms underlying this differential stability remained unclear, these findings indicated that cryoprotectants exerted selective, structure-dependent stabilization effects rather than providing uniform protection across the proteome, which emphasized the critical need to consider biomolecular heterogeneity when designing cryopreservation protocols. Multibatch validation further confirmed the good reproducibility of the optimized CM2 formulation in nanoparticle concentration and total protein content. Moreover, long-term storage detection verified that the core cytokine bioactivity of CM2 could be well maintained after one-year storage at −20°C, further demonstrating that rational compound cryoprotectant formulation could comprehensively improve the structural stability and bioactivity retention of MSC-CM during lyophilization and long-term preservation.

Notably, independent cryoprotectant-only control groups were established in multiple *in vitro* functional assays to distinguish the direct biological actions of protectants from the inherent reparative bioactivity of MSC-CM. The results confirmed that the inherent biological regulation effect of the two protectants was limited and negligible, and the superior anti-inflammatory and tissue-regenerative effects of CM1 and CM2 were predominantly derived from endogenous bioactive components, including cytokines and EVs within conditioned medium. These findings ruled out the confounding interference of protectants themselves and supported that trehalose and PVP40 likely contributed to preserving the structural integrity and bioactive components of MSC-CM during lyophilization *in vitro*. However, due to the lack of CM0 and cryoprotectant-only groups *in vivo*, the definitive contribution of cryoprotectants to *in vivo* efficacy could not be fully confirmed.

### Possible mechanisms of anti-inflammatory and pro-regenerative effects in Lyo-MSC-CM

Skin wound healing requires precisely coordinated inflammation and proliferation phases. During early inflammation (last ∼3–4 days), macrophages orchestrate immune responses: pro-inflammatory M1 phenotypes dominate initially by releasing ROS and cytokines (e.g., TNF-α, IL-1β) to prevent infection, and anti-inflammatory M2 phenotypes later emerge to resolve inflammation via IL-10 secretion [54]. Evidence indicated that MSC critically modulated this macrophage polarization, suppressed pro-inflammatory cytokines, and enhanced immunoregulatory signals through several key signaling pathways, involving NF-κB pathway and Wnt/β-catenin pathway [55–57]. In the NF-κB pathway regulation, IκB kinase (IKK)-mediated IκBα degradation enabled p50/RelA nuclear translocation, and drove expression of cytokines such as IL-1β, TNF-α, IL-6, IL-10, *etc*. [58], within which IL-1β/TNF-α further amplified NF-κB signaling to enhance vascular permeability and neutrophil recruitment, IL-6 boosted inflammatory signals and initiated transition to proliferative phase, and IL-10 conversely formed a crucial negative feedback loop to restrain excessive inflammation [59]. Concurrently, Wnt/β-catenin pathway exhibited biphasic regulation during the inflammatory phase: early inhibition of excessive inflammation, and later promotion of repair transition [60]. The indirect induction of β-catenin/TCF could promote M2 macrophage differentiation to produce anti-inflammatory factors, while the direct transcriptional activation of β-catenin would induce the expression of FGF2 and VEGF to promote repair transition [61].

Consistent with these mechanisms, CM2 significantly reduced pro-inflammatory cytokines (IL-1β, IL-6, TNF-α) in LPS-stimulated macrophages *in vitro* and elevated IL-10 *in vivo*. We assumed that the anti-inflammatory effect of CM2 arose from the immunoregulatory factors in the contents (e.g., MCP-1) and the feedback on inflammatory environment through the above signaling pathways. Nevertheless, further investigation into the underlying mechanisms is necessary. On the one hand, a sequential priming protocol could be applied to elucidate whether CM exposure could induce a state of trained immunity (wherein macrophages should be pretreated with CM, followed by careful removal of the medium and subsequent stimulation with LPS in a CM-free environment). On the other hand, an in-depth analysis of the molecular mechanisms, involving the expression levels, phosphorylation status, and transcriptional activity of key molecules in critical inflammatory signaling pathways should be conducted to further confirm the related mechanisms.

The proliferative phase constitutes a critical determinant of successful wound healing, characterized by fibroblast-mediated tissue restoration [62]. It has been reported that MSC-Exos enhanced fibroblast functionality through multifaceted mechanisms, including activation of PI3K/Akt signaling for granulation tissue formation [25, 63], VEGF-mediated angiogenesis [64], and MAPK/ERK pathway-driven cellular migration [65]. Among them, PI3K/Akt activation promoted granulation tissue formation through mTOR-mediated protein synthesis and bad-inhibited anti-apoptosis [66]. MAPK/ERK induction enhanced fibroblast migration via MMP expression and cytoskeletal reorganization, facilitated by growth factors (e.g., FGF, TGF-β). VEGF signaling pathway enabled oxygen/nutrient delivery, immune cell trafficking, tissue remodeling, and provided the vascular foundation necessary for skin appendage regeneration in complex wounds [67].

Our findings demonstrated that CM2, with higher preservation efficiency of bioactive nanoparticles and growth factors (e.g., KGF), significantly enhanced fibroblast proliferation and migration in a dose-dependent manner. The observed promotion of migration was particularly aligned with established MAPK/ERK pathway mechanism. Although PI3K/Akt pathway involvement constituted a plausible explanation for CM2’s pro-proliferative effects, direct mechanistic evidence for its specific activation by CM2 remained to be established. Concurrently, our findings demonstrated CM2 significantly enhanced the augmented angiogenesis of CM2, evidenced by more than 4-fold increased CD31+ microvessel density and elevated VEGF levels compared to control, confirming VEGF-signaling-mediated neovascularization. Furthermore, CM2 administration at higher concentrations specifically enhanced the deposition of collagen I and FN1, directly supporting dermal reconstruction and reinforcing structural stability of newly synthesized ECM [68]. The accelerated re-epithelialization and collagen matrix formation observed *in vivo* provided functional validation of CM2’s efficacy in enhancing progression through the proliferative stage of skin wound healing.

With the burgeoning development of cell-based therapies designed to treat pathologies refractory to conventional pharmaceuticals, our primary results provided a new strategy for storage and transport of MSC-derived secretome by inducing cryoprotectants in lyophilization process. Future work should delineate cryoprotectant-cytokine interactions and validate pathway-specific mechanisms in humanized models.

### Limitations

Our findings positioned Lyo-MSC-CM as a scalable, shelf-stable alternative to fresh MSC-CM, particularly coformulated with cryoprotectants to further improve its efficacy. However, three limitations warrant consideration. First, the *in vivo* wound-healing study did not include CM0 or a cryoprotectant-only control group. Although *in vitro* experiments confirmed that cryoprotectants exerted negligible biological effects, the absence of these *in vivo* controls restricts the definitive quantification of cryoprotectant contributions to the overall therapeutic efficacy. Second, although the lyophilization process for the conditioned medium demonstrated enhanced stability and practical applicability through dual cryoprotectant optimization, critical parameters including the precise bioactive constituents, the specific effects on exosome/growth factor stability, the storage condition, and maximum shelf life require rigorous quantification to define clinical translation parameters. Third, while *in vitro* assays revealed concentration-dependent bioactivity, optimal therapeutic dosing *in vivo* must be standardized across preclinical models to ensure reproducibility. Fourth, rehydrated Lyo-MSC-CM was directly applied to open wounds as a low-viscosity PBS solution without biomaterial carriers. This aqueous formulation shows limited local retention and rapid clearance, which may lead to mild dosage fluctuations and restrict sustained therapeutic activity at the wound site. Future studies will further optimize the lyophilized formulation, validate *in vivo* controls, establish more reliable *in vitro*–*in vivo* dose translation, and explore suitable delivery carriers to improve local retention to support clinical translation.

## Conclusions

By systematically interrogating cryoprotectant-enhanced lyophilization, this study advanced Lyo-MSC-CM as a multifunctional therapeutic platform for skin wound healing. Our findings underscored the critical role of cryoprotectants, including trehalose and PVP40, in maintaining the bioactivity of labile components during freeze-drying, thereby optimizing their anti-inflammatory and pro-regenerative functions. These results provided novel insights into the formulation of lyophilized CM, and established a direct correlation between cryoprotectant-mediated stabilization and enhanced wound healing outcomes, aiming to advance acellular regenerative therapies by addressing biomolecule preservation and scalability. Future studies must delineate mechanistic roles of CM constituents (e.g., cytokines, matricellular proteins) and optimize cryoprotectant formulations for clinical-grade production to solidify lyophilized CM as a robust therapeutic modality in regenerative medicine.

## Supplementary Material

rbag140_Supplementary_Data

## Data Availability

Data supporting this study are included within the article.
