# Supplementary material for: Advanced lyophilized mesenchymal stem cell-conditioned medium formulation improves therapeutic efficacy in skin wound repair
Source: Regen Biomater. 2026 Jun 29;13:rbag140. doi: 10.1093/rb/rbag140 (PMC13401446; doi:10.1093/rb/rbag140)
Supplement: rbag140_Supplementary_Data [file rbag140_supplementary_data.docx]

**Supplementary material of**

**An advanced lyophilized MSC-conditioned medium formulation improves therapeutic efficacy in skin wound repair**

Xinjiani Chen^1^, Bailei Li^2,3^, Wenxue Gu^2,3^, Mingjuan Li^1^, Zhen Zhang^2,3^**, Xinjiani Chen^1^*

*^1^ Department of Pharmaceutics, College of Medicine, Jiaxing University, Jiaxing, Zhejiang, 314001,* *P. R. China*

*^2^Department of Biotechnology and Biomedicine, Yangtze Delta Region Institute of Tsinghua University, Zhejiang, 314006, P. R. China*

*^3^Zhejiang Key Laboratory of Multiomics and Molecular Enzymology, Yangtze Delta Region Institute of Tsinghua University, Jiaxing, Zhejiang, 314006, China*

E-mail:

[jiani@zjxu.edu.cn](mailto:jiani@zjxu.edu.cn) (Xinjiani Chen), [libl1213@foxmail.com](mailto:libl1213@foxmail.com) (Bailei Li), [gwxhy14@163.com](mailto:gwxhy14@163.com) (Wenxue Gu), [limingjuan@zjxu.edu.cn](mailto:limingjuan@zjxu.edu.cn) (Mingjuan Li), [zhangzhen@tsinghua-zj.edu.cn](mailto:zhangzhen@tsinghua-zj.edu.cn) (Zhen Zhang)

**
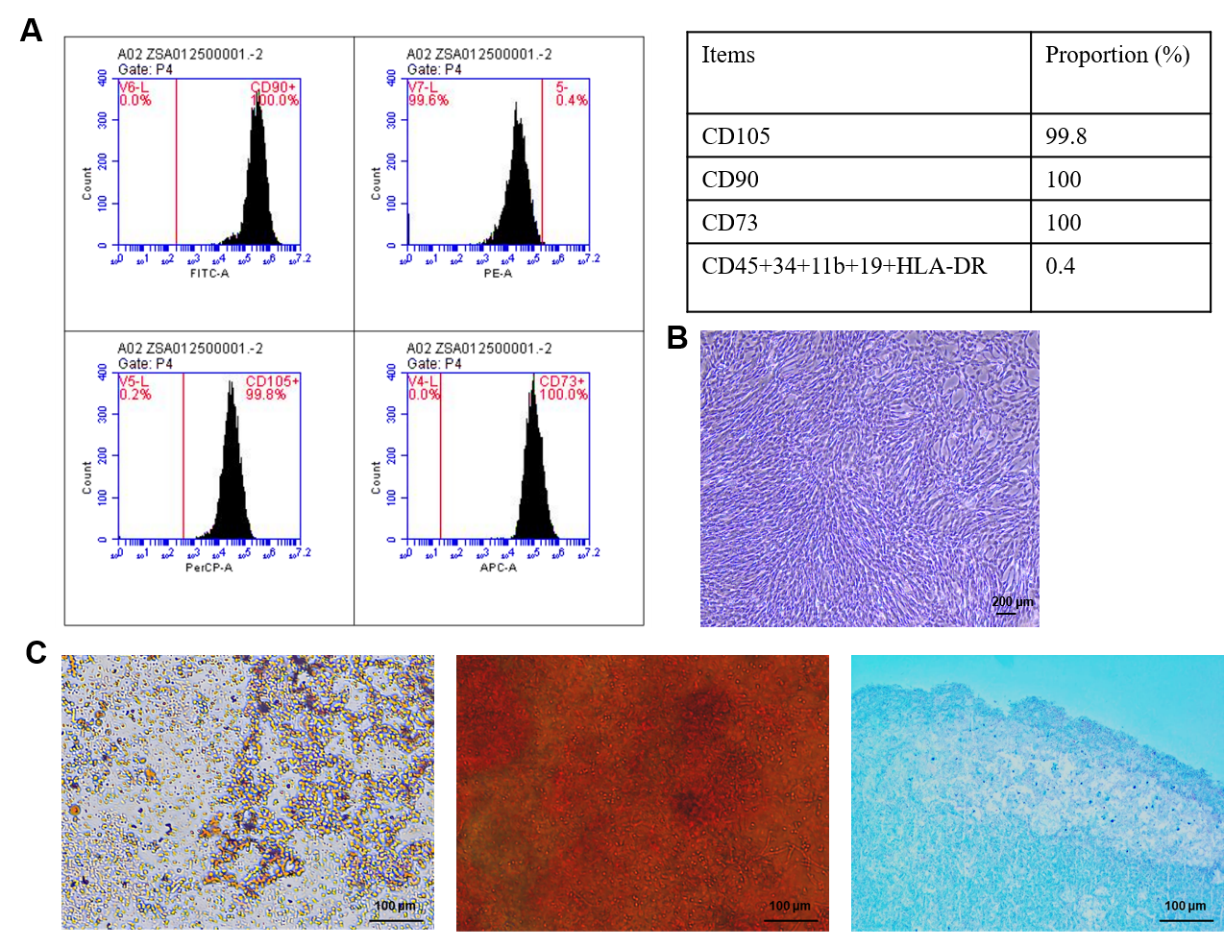
**

**Figure S1.** Identification and characterization of mesenchymal stem cells. **(A)** Identification of surface markers of mesenchymal stem cells by flow cytometry. **(B)** Morphological identification of mesenchymal stem cells by optical microscope. **(C)** Characterization of three lineage differentiation of mesenchymal stem cells. Left: Adipogenic differentiation; middle: Osteogenic differentiation; right: Chondrogenic differentiation.

**Materials and Methods**

Trilineage differentiation assays of MSCs

All trilineage differentiation assays were performed using MSCs at passages 3–5 (P3–P5), which were cultured in a humidified incubator at 37 °C with 5% CO₂ throughout the induction period.

Adipogenic differentiation assay: MSCs at P3–P5 were digested and resuspended as described in the osteogenic differentiation assay, and the cell concentration was adjusted to 2 × 10^4^ cells/mL. A total of 2 mL of cell suspension was seeded into each well of 6-well plates, and the plates were incubated for 24 h to allow cell adherence. The cells were continuously cultured in complete culture medium until they reached 100% confluence. The complete culture medium was discarded, and the cells were washed twice with pre-warmed PBS. Then, 2 mL of adipogenic induction medium (Thermo Fisher, A1007001) was added to each well, and the medium was changed every 3 days to maintain consistent induction conditions for 21 days. Then fixed with 4% paraformaldehyde, stained with Oil Red O (C0158S) according to the instructions, and observed lipid droplets under microscope.

Osteogenic differentiation assay: MSCs (P3-P5) were seeded in 6-well plates at 2×10^4^ cells/well, cultured in complete medium until 80% confluence. Then replace with osteogenic induction medium (Thermo Fisher, [A1007201](https://www.thermofisher.cn/order/catalog/product/A1007201)), refresh medium every 3 days, induced for 21 days. Cells were fixed with 70% ethanol, stained with Alizarin Red S (Beyotime, C0148S) according to the instructions, then observed calcium nodule formation under optical microscope.

Chondrogenic differentiation assay: MSCs at P3–P5 were digested and resuspended to make a cell concentration at 5 × 10^5^ cells/mL. A total of 1 mL of cell suspension was added to each well of 12-well plates, and the plates were centrifuged at 1500 rpm for 5 min at room temperature to form compact cell pellets at the bottom of each well. The cells were continuously induced for 28 days by chondrogenic induction medium (Thermo Fisher, [A1007101](https://www.thermofisher.cn/order/catalog/product/A1007201)). At the end of induction, the cell pellets were carefully collected and fixed in 4% paraformaldehyde. The paraffin-embedded cell pellets were cut into 5 μm-thick sections using a microtome. After deparaffinization and rehydration, the sections were incubated with Alcian Blue staining solution (Beyotime, C0153S) according to the instructions and observed under microscope.

**
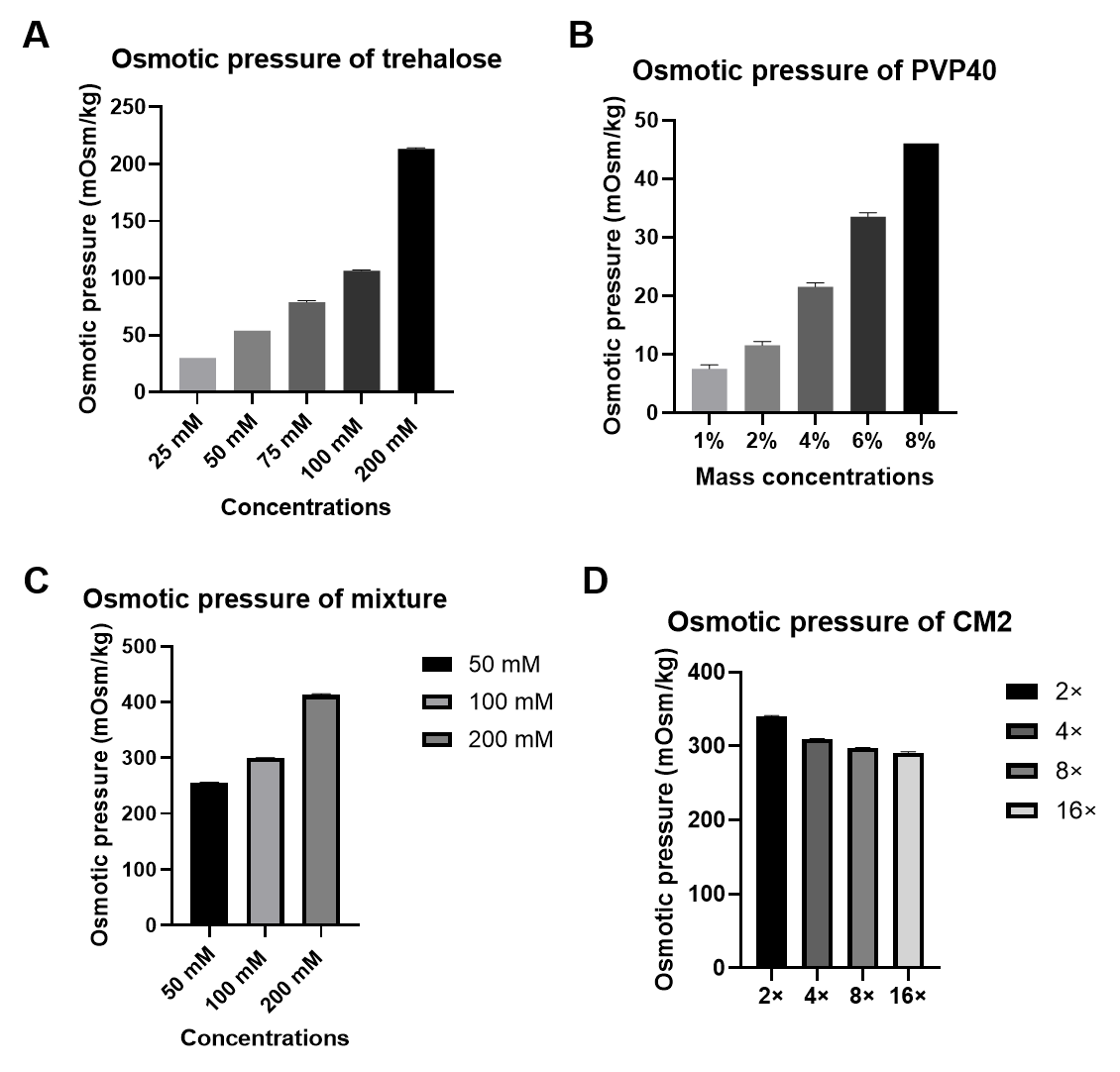
**

**Figure S2.** Osmotic pressure of different concentrations of trehalose and PVP40 dissolved in ultrapure water. **(A)** Osmotic pressure of 25 mM, 50 mM, 75 mM, 100 mM, 200 mM trehalose in ultrapure water. **(B)** Osmotic pressure of 1%, 2%, 4%, 6%, 8% (w/v) PVP40 in ultrapure water. **(C)** Osmotic pressure of different concentrations of trehalose and 2% PVP40 (w/v) dissolved in 1×PBS and ultrapure water mixture (65% 1×PBS with 35% ultrapure water). **(D)** Osmotic pressure of different concentrations of CM2 (2×, 4×, 8×, 16× were defined in the main text in Section 2.9). Data are represented as mean ± SEM of n = 3 independent biological replicates.

**Table S1 Reagent manufacturers and catalog numbers applied in the** **component analysis of conditioned medium**

| Bioactive factors | Sources | Catalog number |
| --- | --- | --- |
| IL-6 | Shanghai Enzyme-linked Biotechnology | ml058097 |
| IL-10 | Shanghai Jianglai Biotechnology | JL19246 |
| TGF-β1 | Shanghai Enzyme-linked Biotechnology | ml022522 |
| MCP-1 | Shanghai Enzyme-linked Biotechnology | ml105321 |
| CXCL-9 | Shanghai Jianglai Biotechnology | JL14160 |
| KGF | Shanghai Enzyme-linked Biotechnology | ml061623 |
| FGF | Thermo Fisher Scientific | KHG0021 |
| VEGF | Hangzhou Lianke Biotechnology | EK183 |
| HGF | Wuhan Elabscience Biotechnology | E-UNEL-H0064 |
| EGF | Wuhan Elabscience Biotechnology | E-EL-H0059 |

**Table S2 Reagent manufacturers and catalog numbers applied in anti-inflammatory experiments**

| Inflammatory factors | Sources | Catalog number |
| --- | --- | --- |
| IL-1β | Wuhan Elabscience Biotechnology | E-EL-M0037 |
| IL-6 | Wuhan Elabscience Biotechnology | E-EL-M0044 |
| TNF-α | Wuhan Elabscience Biotechnology | E-EL-M3063 |

**Table S3 Reagent manufacturers and catalog numbers applied in *in vivo* cytokine profiling**

| cytokines | Sources | Catalog number |
| --- | --- | --- |
| IL-10 | Wuhan Elabscience Biotechnology | E-EL-R0016 |
| FGF | R&D Systems | MFB00 |
| VEGF | Abcam, plc | ab100786 |

**Table S4 Summary of specific functions of cytokines detected in the main text.**

| Inflammation- related cytokines | Main functions |
| --- | --- |
| TNF-α | - **Initiates inflammation**: Activate vascular endothelium to express adhesion molecules (ICAM-1/VCAM-1), facilitating leukocyte extravasation; promote the proliferation and differentiation of macrophages and other immune cells. - **Immunoregulation**: Stimulate immune cells to produce cytokines such as IL-1β, IL-6, and MCP-1 *et al.* |
| IL-1β | - ​**Amplifies inflammation​​**: Activated by NLRP3 inflammasome/caspase-1, inducing fever, vasodilation, and neutrophil recruitment; synergize with IL-6 to promote Th17 differentiation and tissue destruction via MMPs - **Drives acute-phase responses​**​: Stimulates cyclooxygenases (COX-1/COX-2) to produce prostaglandins. |
| MCP-1 (CCL2) | - **Chemotaxis and immune cell recruitment​**​: Bind CCR2 on monocytes, driving migration to inflammation sites via PI3K/Akt signaling; Promote neutrophil differentiation and participate in the recruitment and differentiation of dendritic cells. - **T-cell immune regulation**: Selective induction of initial T cell (Th0) differentiation towards Th2 phenotype; bi-directional regulation of Th1/Th2 balance. - **Inflammation amplification and mediator release**: Activate monocytes/macrophages to release related inflammatory factors; promote the release of oxygen free radicals and cytotoxic enzymes. - Influence the activity of NF-ĸB pathway, Akt signalling pathway, ERK pathway and several others that affect the development and progression of diseases specially in cancer. |
| CXCL-9 (MIG) | - **Immune Cell Recruitment and Activation:** By binding to its receptor CXCR3, CXCL-9 guides T cells and NK cells to sites of inflammation or tumors, enhancing immune surveillance and anti-tumor immunity. - **Angiogenesis inhibition and tumor growth regulation:** CXCL9 can inhibit tumor angiogenesis and growth by recruiting effector immune cells and suppressing pro-tumor pathways**.** |
| IL-6 | - **Acute-phase response:** Stimulate hepatocytes to produce CRP, fibrinogen, serum amyloid A; stimulate activated B cells to differentiate into antibody-producing plasma cells; Modulate T-cell differentiation, especially play important roles in skewing Th17/Treg balance. - **Hematopoietic Effects​:** Promote megakaryocyte maturation in the bone marrow; induce excess VEGF production. |
| IL-10 | - **Anti-inflammatory Effects via Immune Cell Suppression**: Inhibition of antigen-presenting cells (APCs) (reduce expression of MHC class II, costimulatory molecules (CD80/CD86), and pro-inflammatory cytokines such as TNF-α, IL-1β, IL-6, IL-12); Blockade of T-cell activation and promote regulatory T cells (Tregs). - **Activation of Specific Immune Cells**: Enhance survival, proliferation, antibody production of B cells; boost cytotoxic activity, proliferation, and memory responses of CD8⁺ T cells and NK cells​​. - **Regulation of Metabolism and Signaling**: Suppress glycolysis in macrophages via STAT3-DDIT4-mTORC axis to limit inflammation; bind with IL-10R to activate JAK-STAT3 pathway for anti-inflammatory effects. |
| Growth factors | Functions |
| TGF-β1 | - **Modulation of Immune Responses​**: Act as a chemoattractant and promotes pro-fibrotic M2-like phenotypes; promote differentiation of naïve CD4⁺ T cells into regulatory T cells (Tregs); - **Fibroblast/Myofibroblast Activation & Proliferation​​**: Recruit fibroblasts to injury sites at low concentrations; stimulate fibroblast growth via TGF-β1 auto-induction and MAPK pathways; drive α-smooth muscle actin (α-SMA) expression and contractile function via Smad3-AP1 cooperation. - **Regulation of Extracellular Matrix (ECM)** **Dynamics**: Induce ECM synthesis, ​suppress ECM degradation, and stabilize ECM. - Important in tissue protection and repair. |
| KGF | - **Epithelial stimulation**: Stimulate proliferation of epithelial cells when binding to FGFR2-IIIb. - **Tissue repair** **and regeneration**: Accelerate wound healing by promoting re-epithelialization and reducing inflammation in skin, lung, and mucosal injuries; facilitate peripheral nerve regeneration and modulates nociceptive neuron function. - **​Cancer Modulation​**​: Overexpressed in tumors (e.g., breast cancer), driving epithelial-mesenchymal transition (EMT) and progression via autocrine signaling. |
| FGF | - **Cell Proliferation/Migration​**​: Activate RAS/MAPK, PI3K/AKT, and PLCγ pathways to control fibroblast growth, angiogenesis, and tissue remodeling. - **Development & Morphogenesis​**​: Regulate embryonic patterning, organogenesis (e.g., lung, limb), and neural differentiation. - **Cancer Progression**: Stimulate proliferation/invasion in ​**​p**rostate, gastric, and ovarian cancers; induce chemotherapy resistance via FGFR2-IIIb/EMT axis activation. |
| VEGF | - ​**​Angiogenesis​**​: Induce endothelial proliferation, migration, and tube formation via VEGFR-2 activation; critical in development and wound healing. - **Vascular Permeability​**​: Increase microvascular leakage, facilitating nutrient/immune cell extravasation. - ​**Vasculogenesis​​:** Critical for de novo blood vessel formation during embryogenesis. - **Non-Vascular Functions​**: Supportsneuronal survival in the CNS; modulates osteoblast-osteoclast crosstalk; regulate hematopoietic stem cell (HSC) survival and differentiation in bone marrow niches. |
| EGF | - **Wound Healing**: Recruit inflammatory cells; stimulate neutrophil and macrophage activity to release inflammatory factors; accelerate keratinocyte migration/proliferation for re-epithelialization; promote fibroblast proliferation and collagen synthesis; support ECM reorganization via MMP regulation. - **Metabolic Regulation​**​: Modulate gastric acid secretion and nutrient absorption in the digestive system |
| HGF | - Anti-Apoptotic & Anti-Fibrotic​​: Protect endothelial cells from apoptosis and inhibits collagen deposition in fibrotic diseases. - **Regenerative Mitogen​**​: Trigger hepatocyte proliferation and liver regeneration post-injury. - **Morphogenic Activator**​​: Induce epithelial scattering, tubulogenesis, and branching in kidney/lung development via c-MET receptor. - **Tumor Modulation​**​: Promote cancer metastasis by enhancing cell motility, invasion, and angiogenesis. |
| CD31 | - **Angiogenesis Regulation​**​: Modulate endothelial tube formation and vascular integrity; used as an immunohistochemical marker for tumor vasculature. - **Cell Adhesion & Migration​**​: Mediate leukocyte transendothelial migration via homophilic (CD31-CD31) binding at endothelial junctions - **Immunomodulation​**​: Deliver inhibitory signals in T-cells via ITIM domains, regulating immune tolerance and inflammation. |
| (Extracellular Matrix) ECM | Functions |
| FN-1 | - **Promote Cell Adhesion & Migration​​**: Mediate cell attachment to the ECM via integrin receptors; Facilitate embryonic development by regulating adhesion dynamics. - **Enhance Wound Healing**: Promote cell proliferation and migration to damaged sites, accelerating tissue regeneration. - **ECM Assembly**: Bind collagen, heparin, and fibrin to stabilize ECM architecture |
| COL-1 | - **Structural Support**​​: Primary component of bones, skin, tendons, and ligaments to provide tensile strength; essential for skeletal morphogenesis and bone mineralization; interactions with FN-1 (assembled collagen fibers shield FN from cellular traction forces) |
| Elastin | - **Maintain the flexibility and elasticity of blood vessels**: An important substance that constitutes blood vessels; prevent hardening of blood vessel walls; promote the proliferation of cells within blood vessels and assist in vascular repair. - **Structural Support**: Provide low-stiffness elasticity (1,000× less rigid than collagen) to enable reversible deformation at low-to-moderate vascular stretch; synergistic effect with collagen fibers. |

**Abbreviations**

MSCs: Mesenchymal stem cells

CM: Conditioned medium

IL-1β: Interleukin-1beta

IL-6: Interleukin-6

TNF-α: Tumor necrosis factor-alpha

TGF-1β: Transforming growth factor-β

MCP-1: Monocyte chemoattractant protein-1

CXCL-9: C-X-C Motif Chemokine Ligand 9

KGF: Keratinocyte growth factor

HGF: Hepatocyte growth factor

VEGF: Vascular endothelial growth factor

FGF: Fibroblast growth factor

EGF: Epidermal growth factor

FN-1: Fibronectin-1

COL-1: Collagen-1

GAPDH: Glyceraldehyde-3-phosphate dehydrogenase

CD31: Platelet endothelial cell adhesion molecule-1

LPS: Lipopolysaccharides

Dex: Dexamethasone

IGF-1: insulin-like growth factor

NFκ-B: Nuclear factor kappa-B

Wnt: Wingless-related integration site

IKK: Inhibitor of kappa B kinase

PI3K/Akt: Phosphatidylinositol-3-kinase/ protein kinase B

MAPK/ERK: Mitogen-activated protein kinase/ extracellular signal-regulated kinase

MEK: MAP kinase-ERK kinase


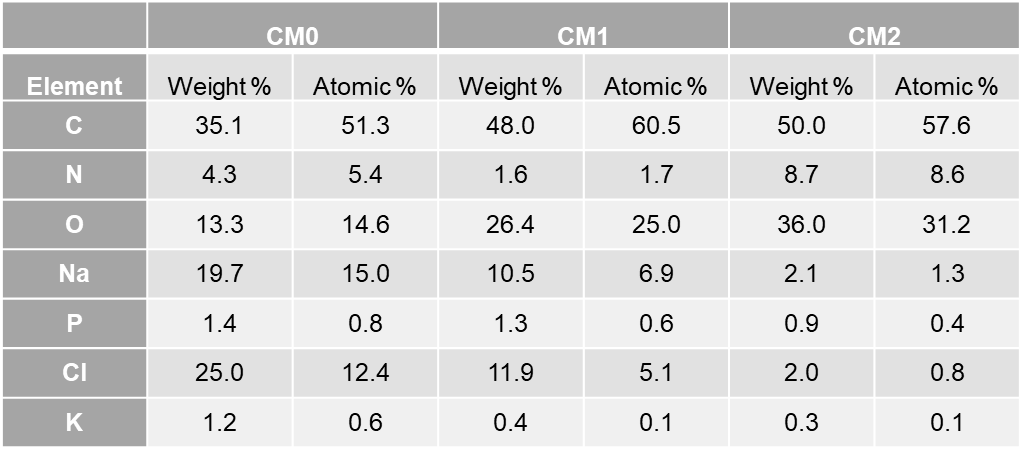


**Figure S3.** Corresponding data analysis of EDS color-coded elemental mapping.


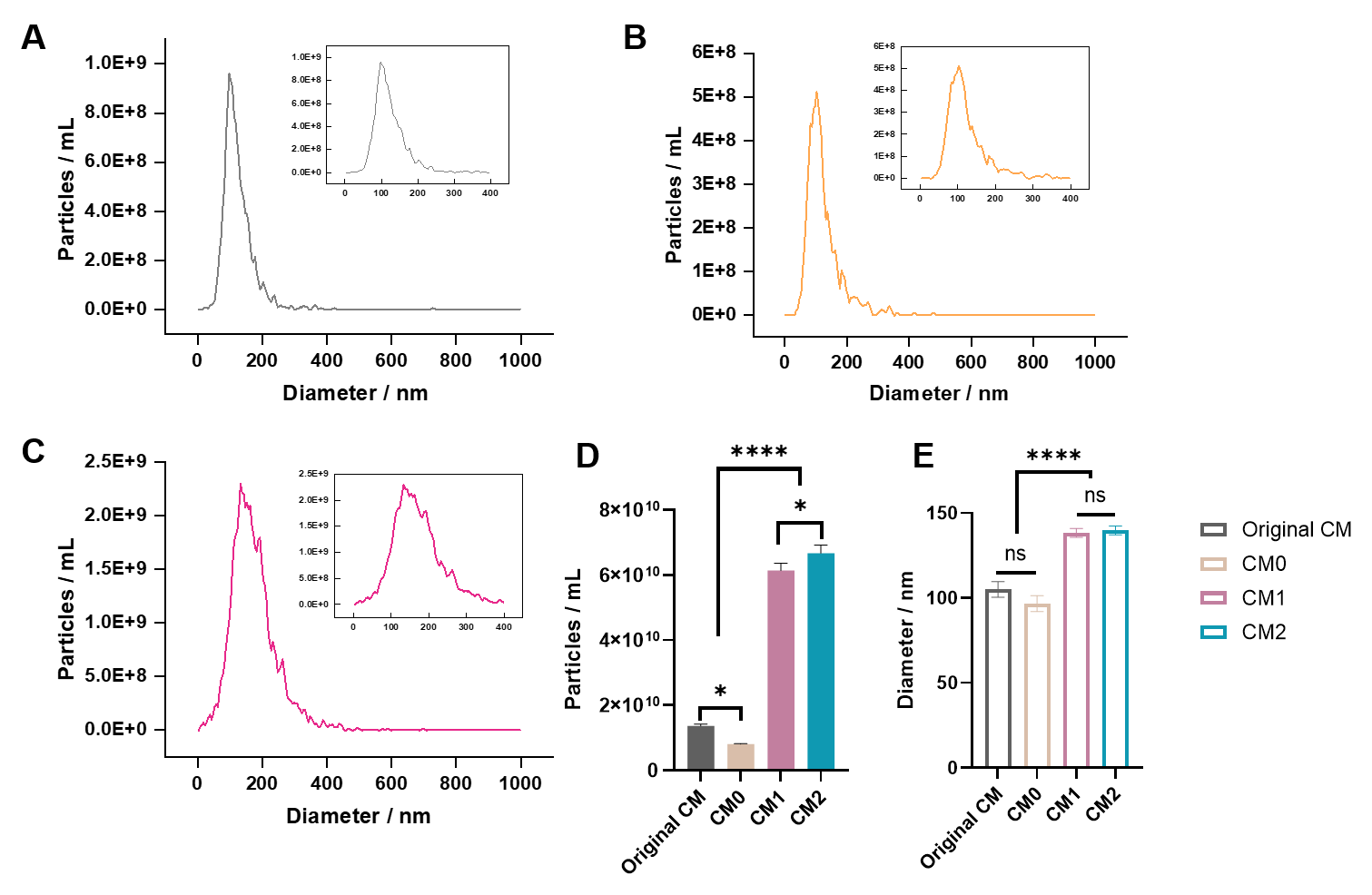


**Figure S4.** Results of Nanoparticle Tracking Analysis (NTA). **(A)** NTA result of Original CM. **(B)** NTA of CM0. **(C)** NTA of CM1. **(D)** Nanoparticle concentration analysis of different CM. **(E)** Diameter analysis of different CM. Data are represented as mean ± SEM of n = 3 independent biological replicates. ns: non-significance, **p* < 0.05, ***p* < 0.01, ****p*<0.001, *****p* < 0.0001.


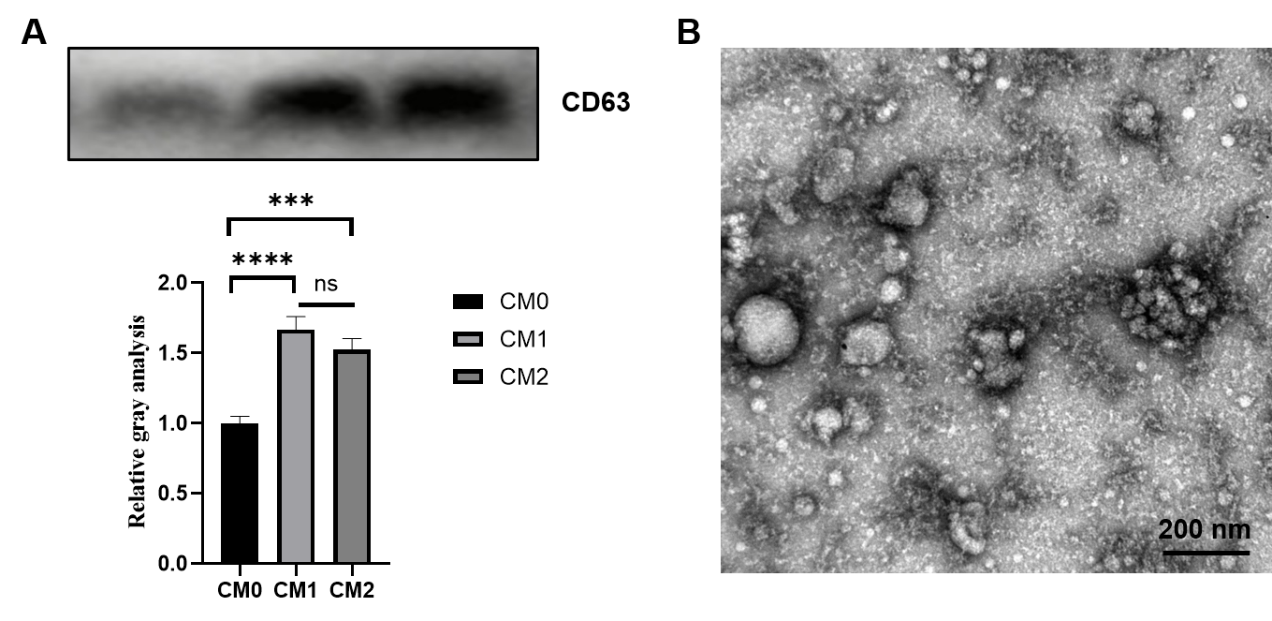


**Figure S5. (A)** Western blotting analysis of CD63 of CM0, CM1, and CM2. The relative gray analysis was normalized by the gray intensity of CM0, data are represented as mean ± SEM of n = 3 per group, ns: non-significance, **p* < 0.05, ***p* < 0.01, ****p*<0.001, *****p* < 0.0001. **(B)** Typical TEM image of CM2.

**Figure S6.** Protein contents of different batches of CM samples analyzed by BCA kits. Data represents n = 5.


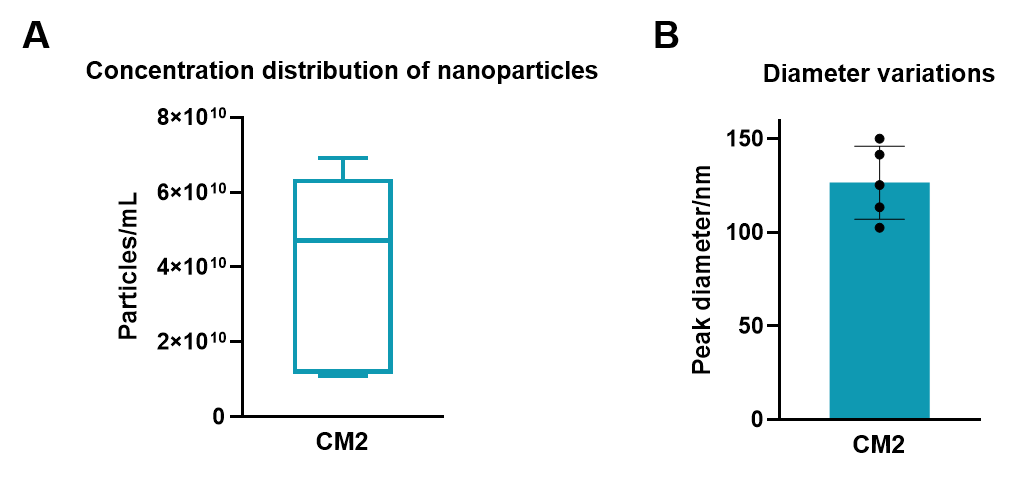


**Figure S7.** Statistical analysis of Nanoparticle Tracking Analysis (NTA) results of CM2. **(A)** concentration of nanoparticles in CM2. **(B)** Peak diameter of CM2. Data represents n = 5.

**Figure S8.** Cytokine contents of the same batch of CM2 detected by ELISA kits timely and after one-year storage at -20 ℃. Data are represented as mean ± SEM of n = 3 per group.

**
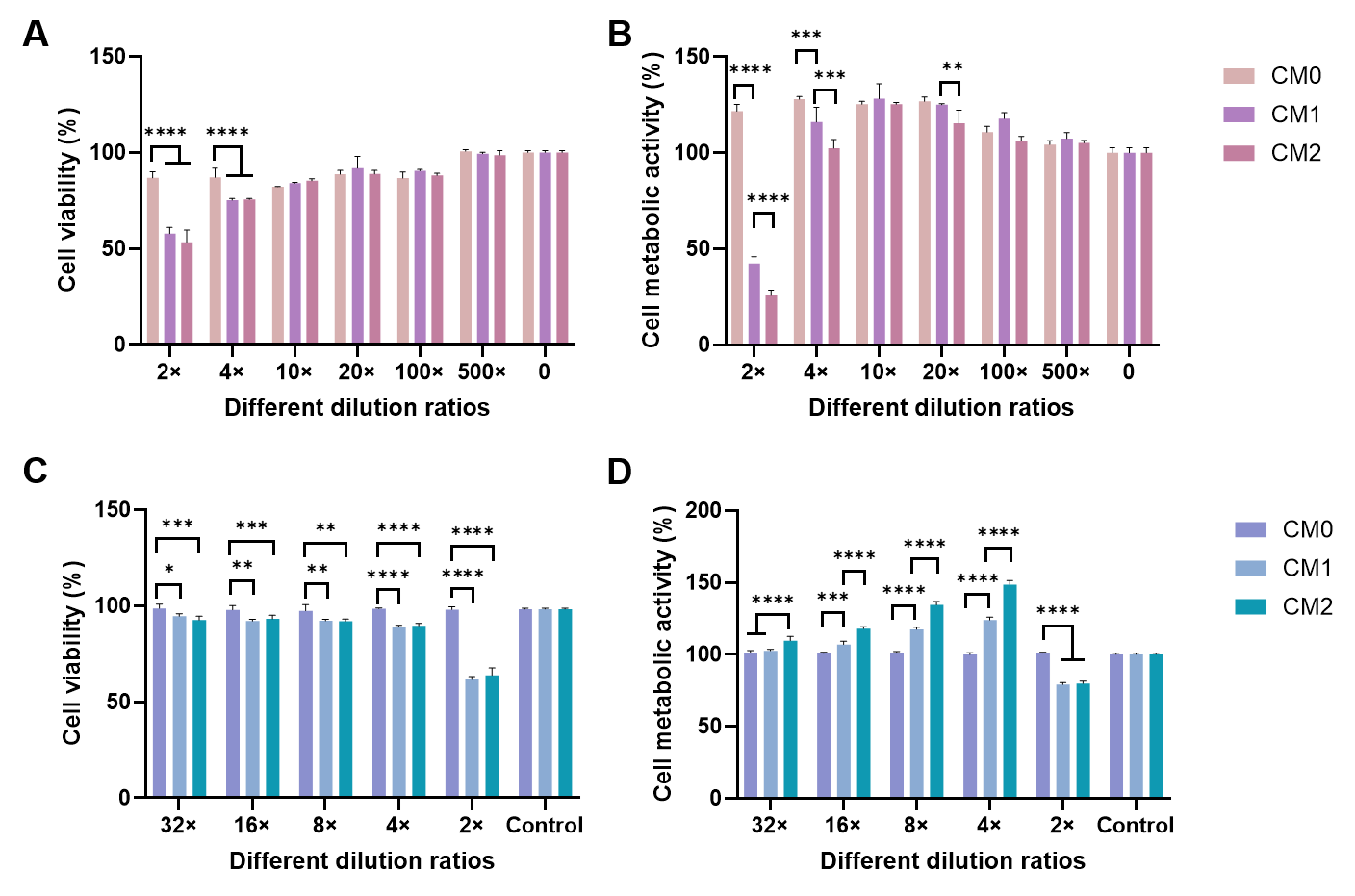
**

**Figure S9.** Effect of lyophilized conditioned mediums on cytotoxicity with CCK-8 kit, another representation of Figure 2. **(A-B)** Effects on L929 fibroblast by comparison under the same concentrations. **(C-D)** Effects on human dermal fibroblasts (HDFs) by comparison under the same concentrations. Data are represented as mean ± SEM of n = 3 per group, ns: non-significance, **p* < 0.05, ***p* < 0.01, ****p*<0.001, *****p* < 0.0001

**Figure S10.** Effect of cryoprotectants on HDF cell viability with CCK-8 kit. data are represented as mean ± SEM of n = 3 per group, ns: non-significance.


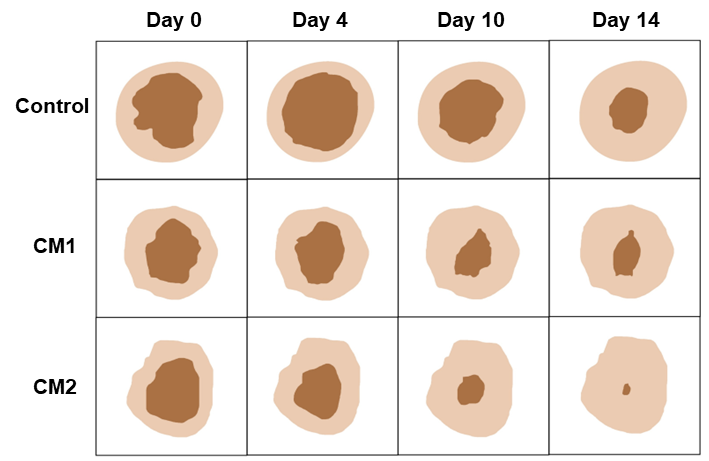


**Figure S11.** Quantitative graph of wound healing over time.
